# Synthesis and reactivity of a mononuclear non-haem cobalt(IV)-oxo complex

**DOI:** 10.1038/ncomms14839

**Published:** 2017-03-24

**Authors:** Bin Wang, Yong-Min Lee, Woon-Young Tcho, Samat Tussupbayev, Seoung-Tae Kim, Yujeong Kim, Mi Sook Seo, Kyung-Bin Cho, Yavuz Dede, Brenna C. Keegan, Takashi Ogura, Sun Hee Kim, Takehiro Ohta, Mu-Hyun Baik, Kallol Ray, Jason Shearer, Wonwoo Nam

**Affiliations:** 1 Department of Chemistry and Nano Science, Center for Biomimetic Systems, Ewha Womans University, Seoul 03760, Korea; 2 Center for Catalytic Hydrocarbon Functionalizations, Institute for Basic Science (IBS), Daejeon 34141, Korea; 3 Department of Chemistry, Korea Advanced Institute of Science and Technology, Daejeon 34141, Korea; 4 Western Seoul Center, Korea Basic Science Institute, Seoul 03759, Korea; 5 Department of Chemistry, Faculty of Science, Gazi University, Ankara 06500, Turkey; 6 Department of Chemistry, University of Nevada, Reno, Nevada 89557, USA; 7 Picobiology Institute, Graduate School of Life Science, University of Hyogo, RSC-UH LP Center, Hyogo 679-5148, Japan; 8 Department of Chemistry, Humboldt-Universität zu Berlin, Brook Taylor Strasse 2, Berlin 12489, Germany; 9 State Key Laboratory for Oxo Synthesis and Selective Oxidation, Lanzhou Institute of Chemical Physics, Chinese Academy of Sciences, Lanzhou 730000, China

## Abstract

Terminal cobalt(IV)–oxo (Co^IV^–O) species have been implicated as key intermediates in various cobalt-mediated oxidation reactions. Herein we report the photocatalytic generation of a mononuclear non-haem [(13-TMC)Co^IV^(O)]^2+^ (**2**) by irradiating [Co^II^(13-TMC)(CF_3_SO_3_)]^+^ (**1**) in the presence of [Ru^II^(bpy)_3_]^2+^, Na_2_S_2_O_8_, and water as an oxygen source. The intermediate **2** was also obtained by reacting **1** with an artificial oxidant (that is, iodosylbenzene) and characterized by various spectroscopic techniques. In particular, the resonance Raman spectrum of **2** reveals a diatomic Co–O vibration band at 770 cm^−1^, which provides the conclusive evidence for the presence of a terminal Co–O bond. In reactivity studies, **2** was shown to be a competent oxidant in an intermetal oxygen atom transfer, C–H bond activation and olefin epoxidation reactions. The present results lend strong credence to the intermediacy of Co^IV^–O species in cobalt-catalysed oxidation of organic substrates as well as in the catalytic oxidation of water that evolves molecular oxygen.

## Abstract (web summary)

Terminal Co^IV^-oxo species are key intermediates in various cobalt-mediated oxidation reactions, but little is known about their chemical properties. Here the authors generate and isolate a mononuclear non-haem Co^IV^-O complex and analyse its structure and reactivity towards a range of catalytic transformations.

High-valent metal–oxo species are implicated as reactive intermediates in the catalytic oxidation of organic substrates and water by a number of enzymes and biomimetic compounds^1^,^2^,^3^,^4^,^5^,^6^. For example, high-valent terminal and bridging iron–oxo species are found to be key oxidizing intermediates in the activation of dioxygen (O_2_) by haem and non-haem iron enzymes; they have now been trapped, spectroscopically characterized and shown to be responsible for a variety of oxidative transformations^1^,^2^,^3^. Similarly, a transient, but not yet isolated, terminal or bridging manganese–oxo intermediate is proposed to be involved in the energy-demanding O–O bond formation step, which is considered to be the most critical part in the oxidation of water to evolve O_2_ in Photosystem II (refs 4, 5, 6). Further, recent synthetic advances have led to the isolation and characterization of several terminal metal–oxo complexes of Fe (refs 7, 8, 9) and Mn (refs 10, 11, 12); detailed reactivity studies, including C–H bond activation and oxygen atom transfer reactions, in conjunction with spectroscopy and theory have helped us to understand how the steric and electronic properties of the metal centres modulate their reactivities. In addition, terminal metal–oxo complexes of Cr, V and Mo have been successfully synthesized in an effort to provide an additional chemical basis for understanding the reaction mechanisms of the metalloenzymes and also to develop artificial oxidation catalysts^13^. Therefore, the chemistry of high-valent terminal metal–oxo species has been well advanced in early transition metals; however, the synthesis and characterization of high-valent terminal metal–oxo complexes of late transition metals, such as Co, Ni and Cu, have remained a long-standing challenge.

The terminal oxo ligands are strong *π*-electron donors, so they readily form stable high-valent metal–oxo complexes with early transition metals. However, for late transition metals with high *d*-electron count, the bond order of the metal–oxo bond decreases in tetragonal symmetry owing to the occupancy of the M–O π* antibonding orbitals, making the terminal metal–oxo complex unstable. Accordingly, isolation of terminal late transition metal–oxo (LTMO) species to the right of group 8 (also known as the oxo wall for *C*_4v_ symmetry)^14^,^15^,^16^ has only been achieved for heavy transition metals like iridium^17^ and platinum^18^ in ligand fields that are not tetragonal. Nevertheless, owing to their importance in a plethora of oxygen-dependent processes, inorganic compounds containing terminal metal–oxo moieties have been discussed or sought for this curious class of LTMOs^19^, which are thought to be reactive intermediates in a number of oxygen-dependent processes. Of particular relevance to this study are the cobalt(IV)–oxo species, which have also been proposed as reactive intermediates in a number of cobalt-mediated oxidation reactions^20^,^21^,^22^,^23^,^24^,^25^,^26^,^27^. In particular, for catalytic oxidation of water to give molecular oxygen, a topic of intensive research aimed at developing artificial photosynthesis and efficient water splitting catalysis, *ex situ* electron paramagnetic resonance (EPR)^28^, X-ray absorption^29^ and time-resolved Fourier-transform infrared^30^ spectroscopic methods have provided direct evidence for the involvement of terminal and bridging cobalt(IV)–oxo species as key intermediates in the water oxidation reactions. However, the proposed cobalt(IV)–oxo intermediates are short lived and highly reactive in most cases, thus making it difficult to study their chemical and physical properties in the catalytic cycles of the cobalt-based oxidation catalysts.

Recently, some of us demonstrated the existence of fleeting LTMO cores in solution by trapping them in presence of redox-inactive metal ions (for example, Sc^3+^) as their Lewis-acid adducts; for example, the intermediate-spin (*S*=3/2) (ref. 31) and low-spin (*S*=1/2) (ref. 32) Co^IV^–O–Sc^3+^ species. However, Borovik and co-workers have suggested an alternative assignment for the former complex invoking a {Co^III^–OH–Sc^3+^} core^33^, thereby refuting the claim of the Co^IV^–O–Sc^3+^ core. Thus, the existence of high-valent cobalt–oxo species and LTMOs is still controversial and remains elusive^19^.

We report herein a photocatalytic generation of a mononuclear non-haem Co^IV^–O complex, [(13-TMC)Co^IV^(O)]^2+^ (**2**) [13-TMC=1,4,7,10-tetramethyl-1,4,7,10-tetraazacyclotridecane], by irradiating its starting cobalt(II) complex, [(13-TMC)Co^II^(CF_3_SO_3_)]^+^ (**1**), in the presence of [Ru^II^(bpy)_3_]Cl_2_ (bpy=2,2′-bipyridine) as a photosensitizer, Na_2_S_2_O_8_ as a sacrificial electron acceptor, and water as an oxygen source. The Co^IV^–O species, **2**, with a significantly higher stability was also synthesized independently by reacting **1** with iodosylbenzene (PhIO) in organic solvent (that is, acetone), and this metastable intermediate was characterized by a commensurately extensive and convincing ensemble of spectroscopic techniques. In particular, the resonance Raman spectroscopy has been employed to provide conclusive evidence for the presence of a terminal Co–O bond in **2**. The reactivities of the Co^IV^–O complex have also been demonstrated in intermetal oxygen atom transfer (OAT), C–H bond activation, and olefin epoxidation reactions. Theoretical studies reveal an unusual bonding situation in **2**, where the *d*(*x*^2^–*y*^2^) orbital is situated lower in energy than the *d*(*z*^2^) orbital. This allows the formation of a full Co–O *π*-bond, even in a tetragonal ligand-field, thereby, leading to the stabilization of the Co^IV^-O core in **2** without violating the oxo wall^14^,^15^,^16^. Thus, our present findings support the involvement of high-valent Co^IV^–O species as reactive intermediates in many important catalytic reactions, such as the oxidation of hydrocarbons and water (Fig. 1).

## Results

### Generation and characterization of a Co^IV^–O complex

The starting cobalt(II) complex, [Co^II^(13-TMC)(CF_3_SO_3_)]^+^ (**1**), was synthesized and characterized by a number of spectroscopic techniques, such as ultraviolet–visible absorption spectroscopy, EPR spectroscopy, electrospray ionization mass spectrometry (ESI-MS) and X-ray crystallography (Supplementary Figs 1 and 2; Supplementary Tables 1 and 2). Interestingly, photoirradiation (>420 nm) of a solution containing **1** (1.0 mM), [Ru^II^(bpy)_3_]^2+^ (5.0 × 10^−2^ mM) and Na_2_S_2_O_8_ (10 mM) in a solvent mixture of acetone and water (*v*/*v*=1:1) at −20 °C under an Ar atmosphere resulted in the formation of a green intermediate (**2**) with a maximum ultraviolet–visible absorption band at 625 nm within 1 min (Fig. 2a). The X-band EPR spectrum of **2** shows signals at *g*=6.5 and 2.02, confirming the spin-quartet (*S*=3/2) ground state (see below). The intermediate **2** was not stable and decayed within 4 min under the reaction conditions. Notably, a faster decay of the intermediate **2** was observed when the irradiation was stopped (Supplementary Fig. 3b); **2** can be regenerated upon exposing the decayed solution to visible light (Supplementary Fig. 3c). The formation of **2** was not observed in the absence of **1**, a photosensitizer, a sacrificial electron acceptor, or visible-light irradiation, suggesting that all these components are necessary for the photocatalytic generation of **2** (Supplementary Fig. 4 for the proposed mechanism of the photo-induced generation of **2**).

Further characterization of **2** (for example, resonance Raman (rRaman) and reactivity studies), however, proved to be difficult due to the instability of the intermediate under the above photocatalytic conditions (vide supra) (Supplementary Fig. 3). Therefore, we attempted to synthesize **2** through more standard methods for the synthesis of metal–oxo complexes using artificial oxidants, such as PhIO, peracids and hydroperoxides^7^,^8^,^9^,^10^,^11^,^12^. Interestingly, among the examined oxidants, we were able to obtain **2** with a significantly higher stability when PhIO was used as a terminal oxidant in the presence of a small amount of acid; the formation of **2** was not clean in the absence of acid due to the longer formation time and the less stability of **2**. Addition of PhIO (3 equiv.) to a solution of **1** (2.0 mM) in the presence of triflic acid (CF_3_SO_3_H, HOTf; 1.2 equiv. to **1**) in acetone at −40 °C immediately generated an EPR-silent transient intermediate (**3**), which was then gradually (∼2 min) converted to a green intermediate (**2**) with a maximum ultraviolet–visible absorption band at 625 nm and a shoulder at 420 nm (Fig. 2b; see also Supplementary Fig. 5 for EPR). Notably, the ultraviolet–visible spectra of the solutions of **2** generated by photochemical (equation (1)) and chemical (equation (2)) conditions differ only in the 400–500 nm region (Supplementary Fig. 6a), which we attribute to the presence of residual [Ru^II^(bpy)_3_]^2+^ and [Ru^III^(bpy)_3_]^3+^ species in solutions of **2** obtained under photocatalytic conditions. In addition, EPR spectra of the two solutions are also identical (Supplementary Fig. 6a, inset), further confirming that the same product **2** is obtained by chemical and photochemical oxidations of **1**. Complex **3** is tentatively assigned to a cobalt–iodosylbenzene adduct complex, [(13-TMC)Co^III^–OIPh]^3+^, with a spin triplet ground state that presumably undergoes O–I bond cleavage to yield **2** containing a Co^IV^–O core. Although the transient nature of **3** prevented a detailed spectroscopic characterization, an unambiguous assignment of the metastable intermediate **2** (*t*_1/2_∼3 h at −40 °C) as a terminal Co^IV^–O species was possible by employing a variety of spectroscopic techniques, such as coldspray ionization time-of-flight mass spectrometer (CSI-TOF MS), rRaman, EPR, X-ray absorption spectroscopy (XAS) and computational methods.


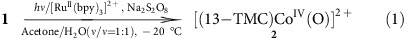



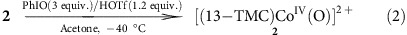


The CSI-TOF MS of **2** exhibits a prominent ion peak at *m*/*z* 466.1 (Fig. 2c), whose mass and isotope distribution patterns correspond to [(13-TMC)Co(O)(CF_3_SO_3_)]^+^ (calcd *m*/*z* 466.1). Upon introduction of ^18^O into **2** using PhI^18^O, two mass unit shift from *m*/*z* 466.1 to 468.1 was observed (Fig. 2c, inset), indicating that **2** contains an oxygen atom derived from PhIO.

The rRaman spectrum of **2**, upon *λ*_ex_=413.1 nm excitation in acetone at −40 °C, exhibits a vibration band at 770 cm^−1^, which shifts to 736 cm^−1^ upon ^18^O-labelling of **2** (Fig. 2d). The observed isotopic shift of Δ*ν*=34 cm^−1^ upon ^18^O-substitution matches with the calculated value of Δ*ν*=34 cm^−1^, as expected for a diatomic Co–O oscillator. It is of interest to note that the Co–O stretching frequency of 770 cm^−1^ for **2** is lower than the Fe–O stretching frequencies of an Fe^IV^=O complex bearing the same supporting ligand, [(13-TMC)Fe^IV^(O)]^2+^ (that is, *ν*(Fe–O)=833 cm^−1^)^34^, and other mononuclear non-haem Fe^IV^=O complexes (that is, *ν*(Fe–O)=820–850 cm^−1^)^35^. Notably, the observed differences are larger than expected from differences in the reduced masses (*μ*) of Fe–O (*μ*=12.44) and Co–O (*μ*=12.58) alone, suggesting that the Co–O bond order of **2** is lower than that of the non-haem Fe^IV^=O complexes, including [(13-TMC)Fe^IV^(O)]^2+^ (see below for EXAFS and computational results and discussions). Although Frei and co-workers proposed the formation of a Co^IV^–O species in a cobalt-oxide (Co_3_O_4_)-catalysed water oxidation reaction and assigned an IR vibrational feature at 840 cm^−1^ to a surface Co^IV^–O site^30^, this current study represents the only example where a diatomic Co–O vibration has been conclusively identified on the basis of isotope labelling studies on a molecular inorganic complex containing a terminal Co^IV^–O core; this helps to clarify the long-existing controversy related to the existence of such cores in coordination complexes.

### Redox titration of 2 with decamethylferrocene

The EPR spectrum of **2** reveals a broad signal of rhombic symmetry, with effective *g* values of *g*_⊥_^eff^=6.5 and *g*_||_^eff^=2.02 and no resolvable ^59^Co hyperfine feature (Supplementary Fig. 7), thereby indicating an *S*=3/2 ground state (Supplementary Fig. 7 for the EPR simulation of **2**). By carrying out titration experiments with a one-electron reductant such as decamethylferrocene (Me_10_Fc), we were able to assign the oxidation state of +4 for the Co ion in **2**. Upon addition of 0.5 equiv. of Me_10_Fc to **2**, the EPR signal in the reaction solution was reduced to one half of the initial intensity and it disappeared completely with the subsequent addition of another 0.5 equiv. of Me_10_Fc (Supplementary Fig. 8a). In these reactions, the signal corresponding to the decamethylferrocenium ion (Me_10_Fc^+^) appeared at *g*=4.4 and 2.00 (Supplementary Fig. 8a,c)^36^. These results are consistent with the reduction of **2** by 1 equiv. of Me_10_Fc, resulting in the formation of a Co^3+^ species with the concurrent formation of Me_10_Fc^+^, as shown in equation (3) (Supplementary Fig. 9 for product analysis by ESI-MS). Further reduction of the Co^3+^ species by adding another 0.5 equiv. of Me_10_Fc resulted in the reappearance of an EPR signal, whose feature was significantly different from that of the spectrum of **2**. The intensity of the new EPR signal became maximum upon addition of 1.0 equiv. of Me_10_Fc to the solution of the Co^3+^ species (Supplementary Fig. 8b). These results demonstrate that the Co^3+^ species was further reduced by Me_10_Fc to form Co^2+^ and Me_10_Fc^+^, as shown in equation (4). Taken together, the formal oxidation state of +4 for the Co ion in **2** was determined by carrying out the titration experiments with one-electron reductant, Me_10_Fc.


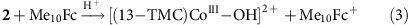



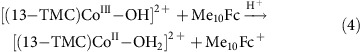


### Cobalt K-edge X-ray absorption spectroscopy of 2

Cobalt K-edge X-ray absorption spectroscopy was then performed to define the structure of **2**. The extended X-ray absorption fine structure (EXAFS) region of the Co K-edge X-ray absorption spectrum was best modelled with cobalt contained in a five coordinate N/O coordination environment with one short Co–O bond at 1.72 Å, and four longer Co–N bonds at an average distance of 2.02 Å (Fig. 3b; see also Supplementary Table 3). In addition, three additional shells containing Co–C scattering pathways were included (4 Co–C at 2.68 Å, 3 Co–C at 2.99 Å, and one Co–C at 3.26 Å). Compared to the known Fe^IV^=O compounds, the EXAFS derived Co–O bond length of **2** is ∼0.07 Å longer than the reported Fe–O bond lengths (∼1.65 Å)^37^. This elongation of the metal–oxo bond in **2** is consistent with the reduced bond order of the Co^IV^–O moiety relative to the Fe^IV^=O moiety, which lends additional support for the structural formulation of **2**.

To better define both the coordination environment about the cobalt center of **2** and its oxidation state, we performed a detailed analysis of the X-ray absorption near edge structure (XANES) region of the Co K-edge spectrum (Fig. 3a). To define a relative energy shift of the edge, we examined the energy at *A*=0.5 relative to the edge jump. Although not a true edge position, this analysis avoids complications due to the presence of low-energy bound transitions found in the XANES region of **2**. Defined in this manner, the edge position occurs at 7,721.6 eV, which is ∼0.8 eV blue-shifted from the known Co(III)–peroxo complexes (Supplementary Fig. 10). Although this is a smaller shift than expected for a change from Co(III) to Co(IV), it is still consistent with an oxidized cobalt center. The pre-edge region of the Co K-edge spectrum yields a reasonably intense pre-edge peak corresponding to a Co(1*s*→3*d*) transition at 7,710.9 eV. The intensity of this peak is consistent with a square pyramidal compound possessing a short axial bond. The absence of an inversion-centre about cobalt will allow for effective mixing of the Co(4*p*_*z*_) orbital into the Co(3*d*_*z*^2^_), which allows for the Co(1*s*→3*d*) transition to gain intensity through a dipole mechanism. The XANES is thus consistent with the formulation of **2** as a Co^IV^–O-like species in nominal *C*_4v_ symmetry^38^.

**Figure 1:**
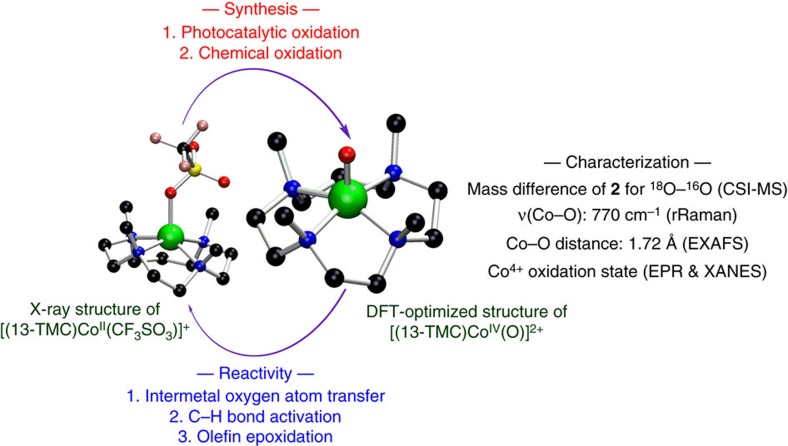
A mononuclear non-haem[(13-TMC)Co^IV^(O)]^2+^complex. Overview of the synthesis of mononuclear non-haem cobalt(IV)–oxo complex [(13-TMC)Co^IV^(O)]^2+^ and its characterization and reactivity.

To gain more insight into the XANES region, we performed TD-DFT (PBE0/def2-tzvp(-f)/ZORA) calculations to obtain theoretical XANES spectra on two previously examined Co(III)–OO(H) species, [(12-TMC)Co^III^(OOH)]^2+^ (12-TMC=1,4,7,10-tetramethyl-1,4,7,10-tetraazacyclododecane)^39^ and [(14-TMC)Co^III^(O_2_)]^+^ (14-TMC=1,4,8,11-tetramethyl-1,4,8,11-tetraazacyclotetradecane)^40^,^41^ and **2** (Fig. 1) using their computationally derived structures. The TD-DFT energies and transition intensities were calibrated to the pre-edge peak of the experimental spectrum of [(12-TMC)Co^III^(OOH)]^2+^, yielding an excellent match to the experimental data (Supplementary Fig. 11). When this calibration was applied to the calculated Co K-edge XAS transitions of both [(14-TMC)Co^III^(O_2_)]^+^ and **2**, we also found an excellent match to the experimental data. Taken together, these XAS results strongly indicate that **2** is best formulated as a square pyramidal Co^IV^–O-like species.

### CASSCF calculations for 2

We then carried out complete active space self consistent field (CASSCF)^42^ calculations at the (11,10)/LANL2DZ^43^,^44^ level of theory, to obtain a more rigorous computational confirmation that the 13-TMC ligand can support a true Co^IV^–O fragment (Supplementary Methods, Supplementary Note 1, Supplementary Tables 4–7 and Supplementary Fig. 12; see also Supplementary Note 2 and Supplementary Tables 8–11 for DFT calculations). Here, a Co–O bond length of 1.666 Å for the quartet state of the Co^IV^–O complex was obtained, which is a bit shorter than the experimentally determined bond length of 1.72 Å (vide supra). However, compared to the previously calculated Fe^IV^=O bond length of 1.618 Å (ref. 34), our calculations predict that the Co–O bond will be 0.05 Å longer than Fe–O, which is in good agreement with the experiments where the bond length difference was estimated to be 0.07 Å (ref. 37). In addition, the Co–O bond-order is calculated to be 1.39, which confirms that there is significant double bond character but to a lesser extent than in the case of the Fe–O analogue. The natural orbitals and their occupation numbers (NOON) are shown in Supplementary Table 4. There are a total of 5.43 electrons in the five frontier orbitals that constitute the non-bonding and antibonding orbital space (Supplementary Table 4). As expected for a quartet Co(IV)-*d* intermediate–spin system^45^, the lowest *d*-based frontier orbital is the non-bonding fully occupied *d*(*xy*) orbital, which has no influence on the Co–O bonding. The remaining three electrons are distributed among the two (Co–O)π* and one (Co–N)σ* orbitals, where the latter is the *d*(*x*^2^–*y*^2^) orbital without direct impact on the Co–O bonding. Thus, the MOs determining the Co–O bonding are the lower lying (Co–O)*σ* and (Co–O)*π*-bonding orbitals that are fully occupied and matched by two half-filled (Co–O)π* antibonding orbitals. Notably, the predicted low-energy of the *d*(*x*^2^–*y*^2^) orbital ensures the stability of a tetragonal Co^IV^–O core in an unusual *d*(*xy*)^2^*d*(*xz*,*yz*)^2^*d*(*x*^2^–*y*^2^)^1^*d*(*z*^2^) electronic configuration, which provides a formal metal–oxo bond order of two, very similar to that expected for the corresponding Fe^IV^=O complex. The CASSCF Natural Orbitals are in complete agreement with this qualitative interpretation, although some excited state mixing reduces the actual bond order somewhat. Based on the spectroscopic characterization and CASSCF calculations, we therefore propose with some confidence that a high-valent Co^IV^–oxo intermediate was generated photochemically and chemically and that the Co–O bond in the Co^IV^–oxo species possesses significant double-bond character.

### Reactivity of 2 in intermetal OAT reactions

We have shown previously that the oxo group of mononuclear non-haem Fe^IV^=O complexes can be transferred to other non-haem iron(II) complexes to form their corresponding iron(II) and Fe^IV^=O complexes, respectively^46^. We therefore investigated the intermetal OAT from the mononuclear non-haem Co^IV^–O complex (**2**) to a non-haem iron(II) complex, [(14-TMC)Fe^II^]^2+^, to compare the chemical property of the Co–O moiety of the mononuclear Co^IV^–O complex to those of the well-characterized Fe–O moieties of mononuclear Fe^IV^=O complexes in intermetal OAT reactions. Addition of [(14-TMC)Fe^II^]^2+^ to the solution of **2** in acetone at −40 °C afforded ultraviolet–visible spectral changes (Fig. 4), in which the absorption band at 625 nm due to **2** decreased with the concomitant increase of the characteristic absorption band of [(14-TMC)Fe^IV^(O)]^2+^ at 820 nm (Supplementary Fig. 13 for the product analysis of the reaction solution by ESI-MS)^46^,^47^. In the reaction, the disappearance and appearance rates of the bands at 625 nm for **2** and at 820 nm for [(14-TMC)Fe^IV^(O)]^2+^, respectively, were identical (Fig. 4, inset). In addition, two well-defined isosbestic points were observed at 560 and 705 nm (Fig. 4). First-order rate constants (*k*_obs_), determined by pseudo-first-order fitting of the kinetic data for the decay of **2** at 625 nm or for the formation of [(14-TMC)Fe^IV^(O)]^2+^ at 820 nm increased proportionally with the increase of [(14-TMC)Fe^II^]^2+^ concentration, leading us to determine the second-order rate constant (*k*_2_) of 1.0(1) M^−1^ s^−1^ at −40 °C (Supplementary Fig. 14). These results demonstrate unambiguously that **2** contains an oxo group which can be transferred to an iron(II) complex, [(14-TMC)Fe^II^]^2+^, to give an Fe^IV^=O complex, [(14-TMC)Fe^IV^(O)]^2+^ and a cobalt(II) complex, [(13-TMC)Co^II^]^2+^ (Fig. 5a(i)). Further, the observation that the oxo group of **2** is transferred to the [(14-TMC)Fe^II^]^2+^ complex to form [(14-TMC)Fe^IV^(O)]^2+^ indicates that the oxidizing power of **2** is stronger than that of [(14-TMC)Fe^IV^(O)]^2+^ (refs 46, 48).

### Reactivity of 2 in C–H bond activation reactions

In the oxidation of organic substrates by the Co^IV^–O complex, we first investigated the C–H bond activation of hydrocarbons by **2**. The addition of xanthene to a solution of **2** in acetone at −40 °C resulted in the disappearance of the intermediate with a first-order decay profile (Fig. 6a). The first-order rate constants, determined by pseudo-first-order fitting of the kinetic data for the decay of **2**, increased linearly with the increase of xanthene concentration (Supplementary Fig. 15), giving a second-order rate constant of 1.5 × 10^−1^ M^−1^ s^−1^ at −40 °C. A kinetic isotope effect (KIE) value of 7.9(6) was obtained in the oxidation of xanthene and xanthene-*d*_2_ by **2** (Supplementary Fig. 15). We also determined second-order rate constants in the oxidation of other substrates having different C–H bond strengths (Supplementary Table 12 and Supplementary Fig. 16), showing a linear correlation between the reaction rates and the C–H bond dissociation energies (BDEs) of substrates (Fig. 6b). On the basis of the large KIE value and the good correlation between the reaction rate and BDEs of substrates, we conclude that hydrogen atom (H-atom) abstraction from the C–H bonds of the substrates by **2** is the rate-determining step (r.d.s.) in the C–H bond activation reactions^7^,^8^,^9^,^10^,^11^,^12^.

Product analysis of the xanthene oxidation by **2** revealed the formation of xanthone in 45(3)% yield (Supplementary Table 13). When the reaction was performed with ^18^O-labelled **2** (**2**-^18^O), the oxygen in xanthone was found to derive from the Co^IV^–O species (Supplementary Fig. 17). We also characterized the decay product of **2** in the xanthene oxidation using various spectroscopic methods, such as ultraviolet–visible, EPR and ESI-MS spectroscopies (Fig. 6a and Supplementary Fig. 18), leading us to propose that **2** was converted to the starting [(13-TMC)Co^II^]^2+^ complex (**1**) by transferring its oxygen atom to the organic substrate by an oxygen-rebound mechanism after the H-atom abstraction step^49^. These results demonstrate that **2** acts as a two-electron oxidant during the activation of hydrocarbon C–H bonds (Fig. 5b(i)).

### Reactivity of 2 in olefin epoxidation reactions

The reactivity of **2** was also investigated in olefin epoxidation reactions. Addition of styrene to a solution of **2** in acetone at −40 °C resulted in the disappearance of the characteristic 625 nm band with a first-order decay profile (Supplementary Fig. 19a). The first-order rate constant, determined by pseudo-first-order fitting of the kinetic data for the decay of **2**, increased linearly with increasing styrene concentration (Supplementary Fig. 19b), giving a second-order rate constant of 3.8 × 10^−3^ M^−1^ s^−1^. A KIE value of 1.0(1) was obtained in the oxidation of styrene and styrene-*d*_8_ by **2** (Supplementary Fig. 19b), indicating that no H-atom abstraction is involved in the r.d.s. of the styrene oxidation by **2**, as is often found to be the case for the mononuclear non-haem metal(IV)–oxo-mediated epoxidation reactions^50^. Similarly, second-order rate constants were determined for the oxidation of other aromatic olefins having different oxidation potentials (Supplementary Table 14 and Supplementary Fig. 20), affording a linear correlation between the reaction rate and the oxidation potentials (*E*_ox_) of the substrates with a slope of −0.94 (Fig. 6c). The latter result demonstrates that **2** possesses an electrophilic character, as expected for the oxidation of olefins by metal–oxygen complexes^51^,^52^,^53^,^54^.

Product analysis of the reaction solution of the styrene epoxidation by **2** revealed the formation of styrene oxide as the major product (Supplementary Table 15). In addition, based on the ^18^O-labelling experiment performed with **2**-^18^O (70(3)% ^18^O), the oxygen atom in the epoxide product (68(3)% ^18^O) was found to derive from the Co–oxo group (Supplementary Fig. 21). It is notable that the epoxidation of *cis*- and *trans*-stilbenes by **2** yielded *cis*- and *trans*-stilbene oxides, respectively (Supplementary Table 15), implying that the epoxidation reaction by **2** is highly stereospecific. The decay product of **2** in the epoxidation of styrene was also analysed using various spectroscopic methods, such as ultraviolet–visible, EPR and ESI-MS spectroscopies (Supplementary Figs 19a and 22); the results led us to conclude that **2** was converted to the starting cobalt(II) complex during the olefin epoxidation reaction. Thus, similar to the C–H activation reaction, **2** acts as a two-electron oxidant during the stereospecific epoxidation of olefins (Fig. 5b(ii)). Finally, the present results demonstrate that mononuclear non-haem Co^IV^–O species are capable of epoxidizing olefins stereospecifically via an OAT mechanism.

## Discussion

Previous computational and *in situ* or *ex situ* spectroscopic studies during catalytic alkane hydroxylation and water oxidation reactions mediated by non-haem cobalt catalysts, including cobalt–oxygen clusters (for example, Co_*x*_O_*y*_), have frequently proposed Co^IV^–O species as the active oxidants that effect the stereospecific C–H activation and O–O bond formation reactions^20^,^21^,^22^,^23^,^24^,^25^,^26^,^27^,^28^,^29^,^30^. However, despite the ubiquity of putative Co^IV^–O intermediates in cobalt-catalysed redox reactions, direct spectroscopic characterization of the Co^IV^–O species under catalytic conditions has been achieved in only extremely rare cases. Further, the presence of Co^IV^–O cores in molecular coordination complexes has been controversially discussed in the literature^31^,^33^. In this work, we succeeded in synthesizing a metastable mononuclear non-haem Co^IV^–O complex, [(13-TMC)Co^IV^(O)]^2+^ (**2**), by PhIO oxidation of the starting cobalt(II) complex, [(13-TMC)Co^II^(CF_3_SO_3_)]^+^ (**1**), that led us to characterize it with various spectroscopic techniques. In particular, the rRaman spectroscopy has been employed in the identification of an ^18^O-sensitive Raman band at 770 cm^−1^, whose observed large ^18^O downshift of 34 cm^−1^, as expected for a diatomic Co–O oscillator, conclusively proves the presence of a Co^IV^–O core in **2**. To the best of our knowledge, this study represents the only example where a terminal metal–oxo vibration has been conclusively identified on the basis of ^18^O-isotope labelling studies on any molecular LTMO cores. In reactivity studies, the intermediate **2** showed a good reactivity in C–H bond activation with a large deuterium KIE value, which provides strong evidence for the involvement of Co^IV^–O intermediates in cobalt-mediated C–H oxidation reactions. In addition, an intermetal OAT reaction by **2** was demonstrated along with the stereospecific epoxidation of olefins by mononuclear non-haem Co^IV^–O species.

More importantly, the intermediate **2** can also be generated photocatalytically using [Ru^II^(bpy)_3_]Cl_2_ as a photosensitizer, Na_2_S_2_O_8_ as a low-cost sacrificial electron acceptor, and water as an oxygen source. Although this study does not provide a full account for the reaction mechanisms that operate during cobalt-mediated water oxidation reactions, it does provide experimental evidence for the generation of a Co^IV^–oxo species under conditions relevant to water oxidation catalysis. Thus, this work provides a fundamental framework for future work aimed at understanding the nature of the cobalt-based species responsible for performing water oxidation reactions in a synthetic biomimetic system. In addition, our present findings together with the previous reports of the well-characterized Ir–O^17^ and Pt–O^18^ complexes reveal that the LTMOs are fairly widespread and can certainly now be regarded as realistic candidates for intermediates in many important catalytic oxidation reactions.

## Methods

### Synthesis and characterization of **1**

The starting cobalt(II) complex, [Co^II^(13-TMC)(CF_3_SO_3_)_2_] (**1**) [13-TMC=1,4,7,10-tetramethyl-1,4,7,10-tetraazacyclotridecane], was synthesized by reacting Co^II^(CF_3_SO_3_)_2_ with a tetradentate 13-TMC ligand in CH_3_CN under an Ar atmosphere^41^. Co(CF_3_SO_3_)_2_ (1.3 mmol, 464 mg) was added to a solution of 13-TMC (1.0 mmol, 242 mg) in CH_3_CN and the resulting mixture was stirred overnight at ambient temperature. The resulting solution was filtered and added to a large volume of precooled ether. The product was obtained as a purple solid after washing with ether for three times and dried under an Ar atmosphere (521 mg, 87% yield). **1** was characterized by various spectroscopic techniques, such as ultraviolet–visible, electron paramagnetic resonance (EPR) and ESI-MSs, together with X-ray crystallography (Supplementary Figs 1 and 2). It is of interest to note that, according to the EPR spectra, the spin state of **1** in acetone is high-spin (*S*=3/2 Co^II^), whereas that in acetonitrile is low-spin (*S*=1/2 Co^II^) (Supplementary Fig. 2b). Elemental analysis: calculated for C_15_H_30_CoF_6_N_4_O_6_S_2_, C 30.05; H 5.04; N 9.35. Found: C 30.11; H 5.08; N 9.41.

### Photochemical generation of Co^IV^–O species (**2**)

**Figure 2:**
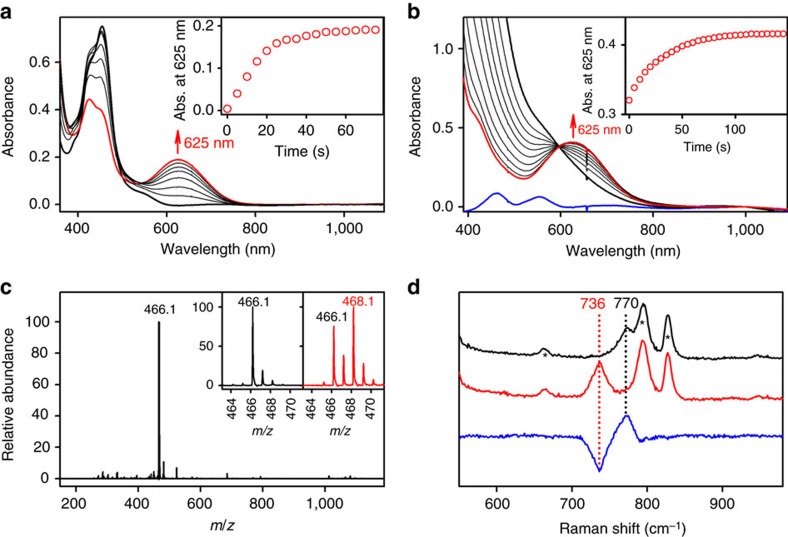
Characterization of 2. (**a**) Ultraviolet–visible spectral changes observed upon photoirradiation (>420 nm) of a deaerated solution of **1** (black line: 1.0 mM) in acetone/H_2_O (*v*/*v*=1/1) with [Ru^II^(bpy)_3_]^2+^ (0.050 mM) and Na_2_S_2_O_8_ (10 mM) at −25 °C. Inset shows the time course monitored at 625 nm due to the formation **2**. (**b**) Ultraviolet–visible spectral changes observed upon addition of PhIO (3 equiv.) to a solution of **1** (blue line; 2.0 mM) in the presence of HOTf (CF_3_SO_3_H, 1.2 equiv.) in acetone at −40 °C. Inset shows the time course monitored at 625 nm during the conversion of **3** (black bold line) to **2** (red line). (**c**) CSI-TOF MS spectrum of **2**. Peak at *m/z*=466.1 corresponds to [(13-TMC)Co(O)(CF_3_SO_3_)]^+^ (calculated *m*/*z*=466.1). Insets show the observed isotope distribution patterns for **2**-^16^O at *m*/*z*=466.1 (left panel) and **2**-^18^O at *m*/*z*=468.1 (right panel). (**d**) Resonance Raman spectra of **2**-^16^O (black line) and **2**-^18^O (red line) obtained upon excitation at 413.1 nm in acetone at −40 °C. Blue line shows the difference between two spectra of **2**-^16^O and **2**-^18^O. The peaks marked with asterisks (*) originate from the solvent.

**Figure 3:**
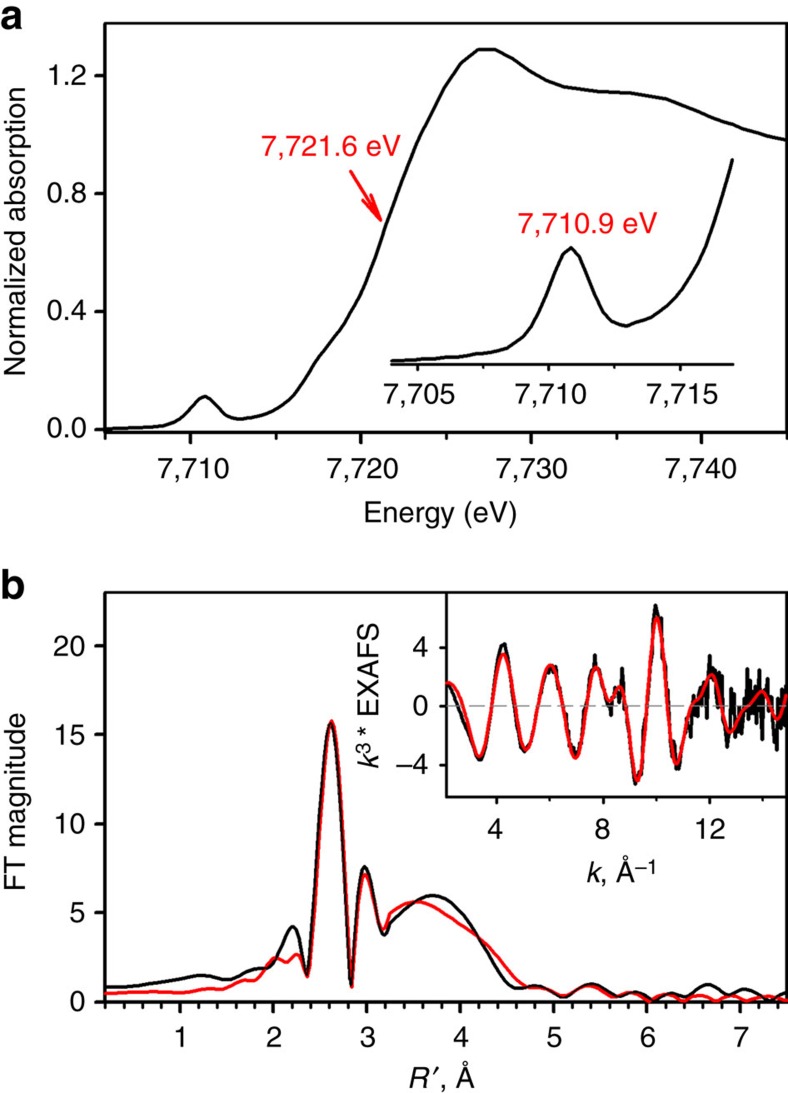
XAS/EXAFS experiments for 2. (**a**) The normalized Co K-edge X-ray absorption spectrum of **2**. (**b**) The non-phase shift corrected magnitude Fourier transform for **2**. Inset shows the corresponding Co K-edge *k*^3^ EXAFS spectrum. Black lines are original data and red lines are the best fits to **2**.

**Figure 4:**
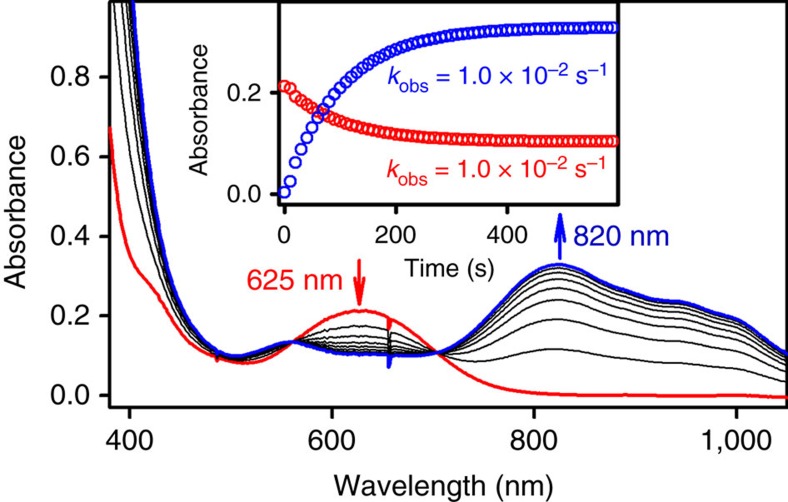
Reactivity of 2 in intermetal OAT. Ultraviolet–visible spectral changes observed in the oxygen atom transfer from **2** to [(14-TMC)Fe^II^]^2+^, showing the formation of [(14-TMC)Fe^IV^(O)]^2+^ (blue line) upon addition of [(14-TMC)Fe^II^]^2+^ (10 equiv.; 10 mM) to a solution of **2** (1.0 mM; red line) in the presence of HOTf (1.2 equiv.) in acetone at −40 °C. The inset shows the time courses monitored at 625 nm for the decay of **2** (red circles) and at 820 nm for the formation of [(TMC)Fe^IV^(O)]^2+^ (blue circles).

**Figure 5:**
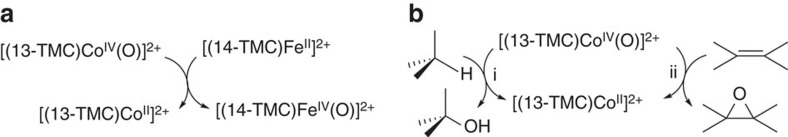
Diversified reactivities of 2. (**a**) Intermetal oxygen atom transfer reaction between [(13-TMC)Co^IV^(O)]^2+^ and [(14-TMC)Fe^II^]^2+^. (**b**) (i) C–H bond activation and (ii) olefin epoxidation by **2**.

The deaerated acetone/water (*v*/*v*=1:1) solution containing Co^II^(13-TMC)(CF_3_SO_3_)_2_ (1.0 mM), [Ru^II^(bpy)_3_]^2+^ (5.0 × 10^−2^ mM) and Na_2_S_2_O_8_ (10 mM) was added into a ultraviolet–visible cuvette. Visible-light irradiation (*λ*>420 nm) with a xenon light source (300 W, MAX-302, Asahi Spectra, Co.) equipped with a 500 nm cut-on filter (MJL Crystek Inc., 500FH90-50) caused the immediate changes in the ultraviolet–visible spectrum that led to the formation of **2**, as evidenced by the appearance of its characteristic band at 625 nm (see Fig. 2a and Supplementary Fig. 3).

### Chemical generation of the Co^IV^–O complex (**2**)

The green intermediate, [(13-TMC)Co^IV^(O)]^2+^ (**2**), was generated by reacting Co^II^(13-TMC)(CF_3_SO_3_)_2_ (2.0 mM) with PhIO (3.0 equiv.; dissolved in 50 μl of trifluoroethanol) in the presence of HOTf (1.2 equiv.) in acetone at −40 °C. The full formation of **2** was confirmed by monitoring ultraviolet–visible spectral changes at 625 nm (Fig. 2b). The ^18^O-labelled [(13-TMC)Co^IV^(O)]^2+^ complex (**2**-^18^O) was prepared by using PhI^18^O in the presence of HOTf (1.2 equiv.) in acetone at −40 °C, PhI^18^O was prepared by mixing PhI^16^O (dissolved in trifluoroethanol) with H_2_^18^O (10 μl). For the resonance Raman experiment (Fig. 2d), **2**-^18^O was prepared by incubating PhIO in the presence of H_2_^18^O (10 μl) prior to reaction with **1** (18 mM) in the presence of HOTf (1.2 equiv.) in an acetone/trifluoroethanol (*v*/*v*=1:1) solvent mixture at −40 °C.

### Instrumentations

Ultraviolet–visible spectra were recorded on a Hewlett Packard Agilent 8453 ultraviolet–visible spectrophotometer equipped with an UNISOKU cryostat system (USP-203; UNISOKU, Japan). CSI-TOF MS spectral data were collected on a JMS-T100CS (JEOL) mass spectrometer equipped with a CSI source. Typical measurement conditions were as follows: needle voltage: 2.2 kV, orifice 1 current: 50–500 nA, orifice 1 voltage: 0–20 V, ring lens voltage: 10 V, ion source temperature: 5 °C, spray temperature: −40 °C. The CSI-TOF mass spectra of **2** and **2**-^18^O were obtained by infusing the reaction solution directly into the ion source through a pre-cooled tube under high N_2_ gas pressure. ESI-MS spectra were collected on a Thermo Finnigan (San Jose, CA, USA) LCQ Advantage MAX quadrupole ion trap instrument, by infusing samples directly into the source at 20 μl min^−1^ with a syringe pump. The spray voltage was set at 3.7 kV and the capillary temperature at 80 °C. Resonance Raman scattering was dispersed by a single polychromator (Ritsu Oyo Kogaku, MC-100DG) and was detected by a liquid-nitrogen-cooled CCD detector (HORIBA JOBIN YVON, Symphony 1,024 × 128 Cryogenic Front Illuminated CCD Detector). Raman spectra were collected with backscattering geometry at an excitation wavelength (*λ*_ex_) of 413.1 nm using a spinning sample cell (NMR tube (5 mm OD)), which was placed in a thermostated quarts dewar at −40 °C by flashing cold nitrogen gas. The laser power at a measuring point in front of a quartz dewar was adjusted to 20 mW. Raman shifts were calibrated using indene (accurate to within ±1 cm^−1^). X-band EPR spectra were taken at 5.0 K using an X-band Bruker EMX-plus spectrometer equipped with a dual mode cavity (ER 4116DM). Low temperatures were achieved and controlled with an Oxford Instruments ESR900 liquid He quartz cryostat fitted with an Oxford Instruments ITC503 temperature and gas flow controller. The experimental parameters for EPR spectra were as follows: Microwave frequency=9.647 GHz, microwave power=1.0 mW, modulation amplitude=10 G, gain=1 × 10^4^, modulation frequency=100 kHz, time constant=40.96 ms and conversion time=81.00 ms. Electrochemical measurements were performed on a CH Instrument (CHI630B) electrochemical analyzer in CH_3_CN/acetone (*v*/*v*=1:1) in the presence of 0.10 M tetra-*n*-butylammonium hexafluorophosphate (Bu_4_NPF_6_) as a supporting electrolyte. A conventional three-electrode cell was used with a platinum working electrode (surface area of 0.3 mm^2^) and a platinum wire as a counter electrode. The platinum working electrodes (BAS) were polished routinely with BAS polishing alumina suspension and rinsed with CH_3_CN before use. The measured potentials were recorded as a function of Ag/AgNO_3_ (0.01 M) reference electrode. All potentials (versus Ag/Ag^+^) were converted to values versus SCE by adding 0.29 V (ref. 55). Elemental analysis was performed on a Thermo Finnigan Italia SpA (Flash EA 1,112) CHN analyzer. 2,5-Bis(5′-tert-butyl-2-benzoxazol-2-yl)thiophene (BBOT) was used as a reference standard. Product analysis was performed with Agilent Technologies 6890N gas chromatograph (HP-5 column, 30 m × 0.32 mm × 0.25 μm film thickness) with a flame ionization detector and Thermo Finnigan (Austin, TX, USA) FOCUS DSQ (dual stage quadrupole) mass spectrometer interfaced with Finnigan FOCUS gas chromatograph (GC–MS). High-performance liquid chromatography (HPLC) analysis was performed on Waters DIOMEX Pump Series P580 equipped with a variable wavelength UV-200 detector and SunFire C18 5 μm column (4.6 mm × 25 mm).

### X-ray structural analysis

Purple coloured single crystals of **1** suitable for X-ray analysis were obtained by slow diffusion of Et_2_O into an acetone solution of **1**. Crystallographic data collections were carried out on a Bruker SMART AXS diffractometer equipped with a monochromator in the Mo Kα (*λ*=0.71073 Å) incident beam. Single crystals of **1** were mounted on a glass fibre tip with epoxy cement. The diffraction data for **1** were collected at 100 K on a Bruker SMART AXS diffractometer equipped with a monochromator in the Mo Kα (*λ*=0.71073 Å) incident beam. The CCD data were integrated and scaled using the Bruker-SAINT software package, and the structure was solved and refined using SHEXTL V 6.12 (ref. 56). The crystallographic data and selected bond distances and angles for **1** are listed in Supplementary Tables 1 and 2.

### EXAFS experiments

Cobalt K-edge X-ray absorption spectroscopic data were collected on the HXMA beamline at the Canadian Light Source (Saskatoon, SA, Canada). Solutions of **2** (2.0 mM in acetone) were injected between Kapton tape windows in aluminium sample holders and quickly frozen in liquid nitrogen. Data were collected at 20 K with sample temperatures maintained using an Oxford liquid He cryostat. Light was monochromatized using a Si(220) double crystal monochromator, which was detuned 50% for harmonic rejection, and focused using a Rh mirror. Spectra were obtained in fluorescence mode using a 32-element solid-state Ge detector with a 3 μm cobalt filter placed between the sample and detector. All spectra were calibrated against the first inflection point of Co-foil, which was simultaneously recorded with the sample. Data were obtained in 10 eV steps in the pre-edge region (7,508–7,700 eV, 1 s integration time), 0.3 eV steps in the pre-edge region (7,700–7,725 eV, 2 s integration time), 1.0 eV steps in the edge region (7,725–7,755 eV, 2 s integration time), 2.0 eV steps in the near-edge region (7,755–8,000 eV, 3 s integration time) and 0.05k steps in the far-edge region (8,000 eV–16.0 Å^−1^, 3 s integration time). To avoid sample photoreduction, the 1 × 1 mm beam spot was moved after every two scans. Total fluorescence counts were maintained under 30 kHz, and a deadtime correction yielded no appreciable change to the data. The reported spectra represent the averaged spectra from eight individual data sets. Prior to data averaging each spectrum and detector channel was individually inspected for data quality. Although data were recorded to 16 Å^−1^, the data were analysed only to 15.0 Å^−1^ owing to noise at high *k*. Data were subsequently processed and analysed as previously reported using EXAFS123 and FEFF 9.4 (ref. 57). Errors to the models are reported as *ɛ*^2^ values (Supplementary Table 3).

### TD-DFT simulation of the Co XAS data

The calculations for simulating the Co XANES data were performed using ORCA v 3.0.3 (ref. 58). Geometry optimizations on **2**, [(12-TMC)Co^III^(OOH)]^2+^ and [(13-TMC)Co^III^(O_2_)]^+^ were performed at the PBE0 level using the def2-tzvp basis set and the ORCA VeryTightSCF convergence criteria. All other parameters were set to the program defaults. TD-DFT calculations were then performed at the PBE0 level using the def2-tzvp(-f) basis set, and the ZORA relativistic approximation. The first 25 spins allowed transitions originating from the Co(1 s) orbital were calculated. Spectra were simulated by applying a Gaussian line shape to each individual transitions (full-width at half-maximum=1.2 eV), and summing the individual transitions. Each calculated transition was blue-shifted by 257.2 eV.

### Kinetic studies

All reactions were run in a 1.0 cm quartz cuvette and followed by monitoring ultraviolet–visible spectral changes of the reaction solutions of **2** in the presence of HOTf (1.2 equiv.) in acetone at −40 °C. The kinetic experiments were run at least in triplicate, and the data reported represent the average of these reactions. Rate constants were determined under pseudo-first-order conditions (for example, [substrate]/[intermediate]>10) by fitting the absorbance changes at 625 nm for the disappearance of **2**. Substrates with varying BDEs^59^, such as 9,10-dihydro-10-methylacridine (AcrH_2_) (73.7 kcal mol^−1^), xanthene (75.5 kcal mol^−1^), 9,10-dihydroanthracene (77.0 kcal mol^−1^), 1,4-cyclohexadiene (78.0 kcal mol^−1^) and fluorene (80.0 kcal mol^−1^), were used in the C–H bond activation reactions by **2** in the presence of HOTf (1.2 equiv.) in acetone at −40 °C. The KIE value for the reaction of **2** and xanthene was determined as the ratio of the *k*_2_ values obtained in the C–H and C–D bond activation reactions of xanthene-*h*_2_ and xanthene-*d*_2_, respectively. The values of *k*_2_′ were obtained by dividing the second-order rate constants *k*_2_ by the number of equivalent target C–H bonds in the substrate. In olefin epoxidation, the reactions of **2** with olefins, such as *trans*-stilbene, *cis*-stilbene, 4-methylstyrene, styrene and 4-chlorostyrene, were investigated in the presence of HOTf (1.2 equiv.) in acetone at −40 °C. Similarly, the KIE value for the oxidation reaction of styrene by **2** was determined as the ratio of the *k*_2_ values of styrene-*h*_8_ and styrene-*d*_8_.

The intermetal oxygen atom transfer from the mononuclear non-haem cobalt(IV)–oxo complex (**2**) to a non-haem iron(II) complex, [(14-TMC)Fe^II^]^2+^, was carried out in acetone at −40 °C. Addition of [(14-TMC)Fe^II^]^2+^ (more than 10 equiv. of **2**) to the solution of **2** afforded ultraviolet–visible spectral changes, in which the absorption band at 625 nm due to **2** decreased with the concomitant increase of the characteristic absorption band of [(14-TMC)Fe^IV^(O)]^2+^ at 820 nm with first-order time profile. In the reaction, the disappearance and appearance rates of the bands at 625 nm for **2** and at 820 nm for [(14-TMC)Fe^IV^(O)]^2+^, respectively, were the same. The first-order rate constants (*k*_obs_), determined by pseudo-first-order fitting of the kinetic data for the decay of **2** at 625 nm or for the formation of [(14-TMC)Fe^IV^(O)]^2+^ at 820 nm increased proportionally with the increase of [(14-TMC)Fe^II^]^2+^ concentration, leading us to determine the second-order rate constant (*k*_2_) of 1.0(1) M^−1^ s^−1^ at −40 °C (Supplementary Fig. 14).

### Product analysis

**Figure 6:**
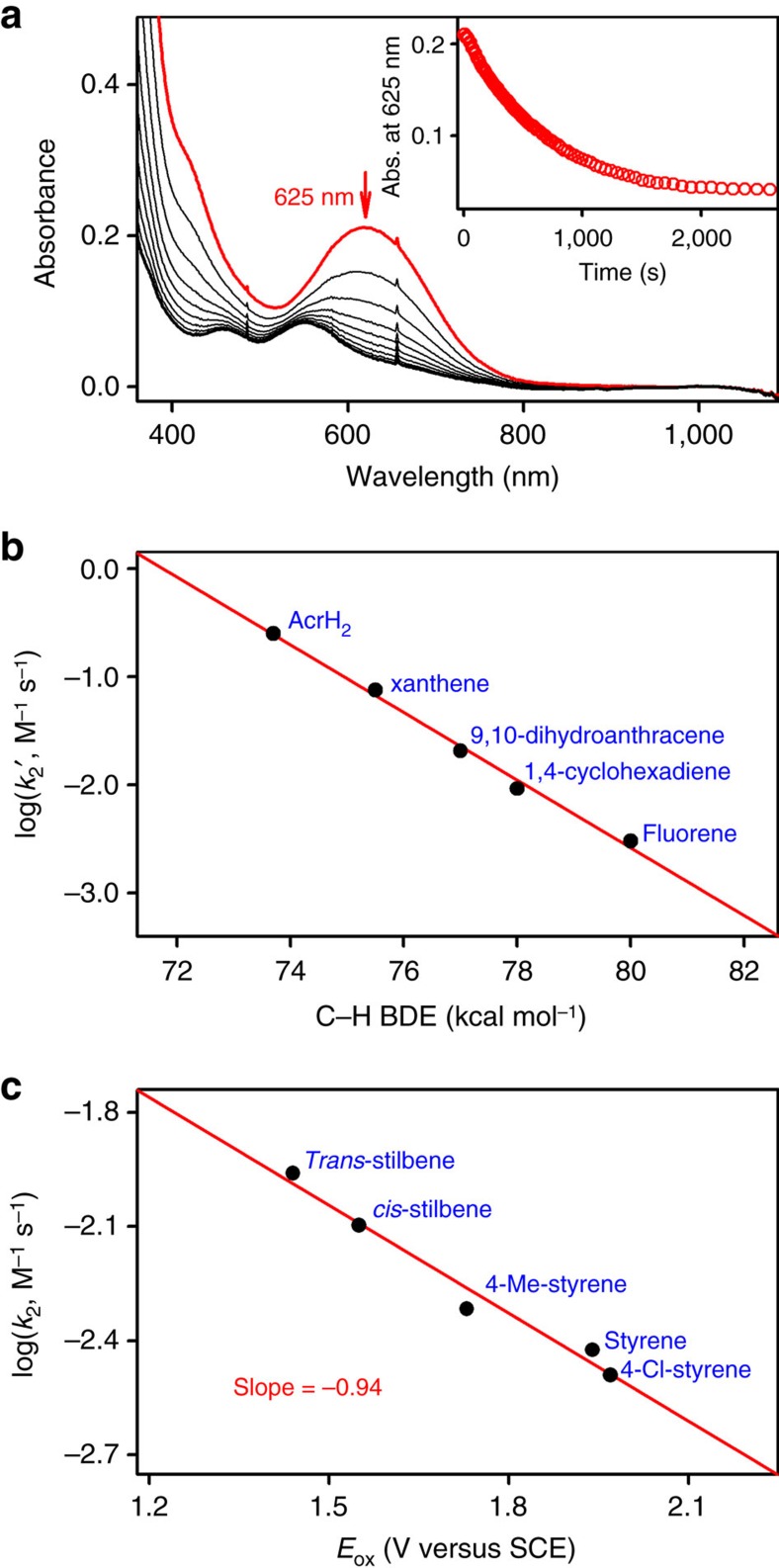
Reactivity of 2 in C–H bond activation and olefin epoxidation. (**a**) Ultraviolet–visible spectral changes observed in the reaction of **2** (1.0 mM) and xanthene (10 mM) in acetone at −40 °C. Inset shows the time course of the decay of 625 nm band associated with **2**. (**b**) Plot of log *k*_2_′ versus C–H BDEs of substrates in the oxidation of substrates by **2**. The *k*_2_' values are obtained by dividing second-order rate constants (*k*_2_) by the number of equivalent target C–H bonds in the substrates. (**c**) Plot of log *k*_2_ for **2** against the *E*_ox_ values of the different olefin substrates.

The cobalt products obtained in the reaction of **2** with xanthene and styrene were characterized by ultraviolet–visible (Fig. 6 and Supplementary Fig. 19a), X-band EPR (Supplementary Figs 18a and 22a) and ESI-MS (Supplementary Figs 18b and 22b) spectroscopic methods. Both EPR and ESI-MS spectra of the final reaction solution of **2** with xanthene and styrene indicate that Co(II) species was formed as a sole product.

Organic products formed in the reactions of **2** with hydrocarbons and olefins were identified by GC and GC–MS methods by comparison of the mass spectra and retention time of the products with that of the authentic samples, and the product yields were determined by comparing the responsive peak areas of sample products against standard curves prepared with known authentic compounds using internal standard decane or nitrobenzene. For *cis*- and *trans*-stilbenes, product yields were determined by HPLC equipped with SunFire C18 5 μm column (4.6 mm × 25 cm). For the ^18^O labelling experiment, the ^16^O and ^18^O compositions in the oxygenated products of xanthene and styrene were analysed by comparing the relative abundances of *m*/*z* values that shifted by two mass units upon incorporation of ^18^O from **2**-^18^O relative to that of ^16^O products.

### Data availability

The X-ray crystallographic coordinates for structures reported in this article have been deposited at the Cambridge Crystallographic Data Centre (CCDC), under deposition number CCDC-1500945. These data can be obtained free of charge via www.ccdc.cam.ac.uk/data_request/cif (or from the Cambridge Crystallographic Data Centre, 12, Union Road, Cambridge CB2 1EZ, UK; fax: (+44) 1223-336-033; or email deposit@ccdc.cam.ac.uk). All other data that support the findings of this study are available within Supplementary Information Files, and are also available from the corresponding authors on reasonable request.

## Additional information

**How to cite this article:** Wang, B. *et al*. Synthesis and reactivity of a mononuclear non-haem cobalt(IV)-oxo complex. *Nat. Commun.* **8,** 14839 doi: 10.1038/ncomms14839 (2017).

**Publisher's note:** Springer Nature remains neutral with regard to jurisdictional claims in published maps and institutional affiliations.

## Supplementary Material

Supplementary Information Supplementary Figures, Supplementary Tables, Supplementary Notes, Supplementary Methods and Supplementary References
