# Supplementary material for: Synthesis and reactivity of a mononuclear non-haem cobalt(IV)-oxo complex
Source: Nat Commun. 2017 Mar 24;8:14839. doi: 10.1038/ncomms14839 (PMC5376677; doi:10.1038/ncomms14839)
Supplement: Supplementary Information — Supplementary Figures, Supplementary Tables, Supplementary Notes, Supplementary Methods and Supplementary References [file ncomms14839-s1.pdf]

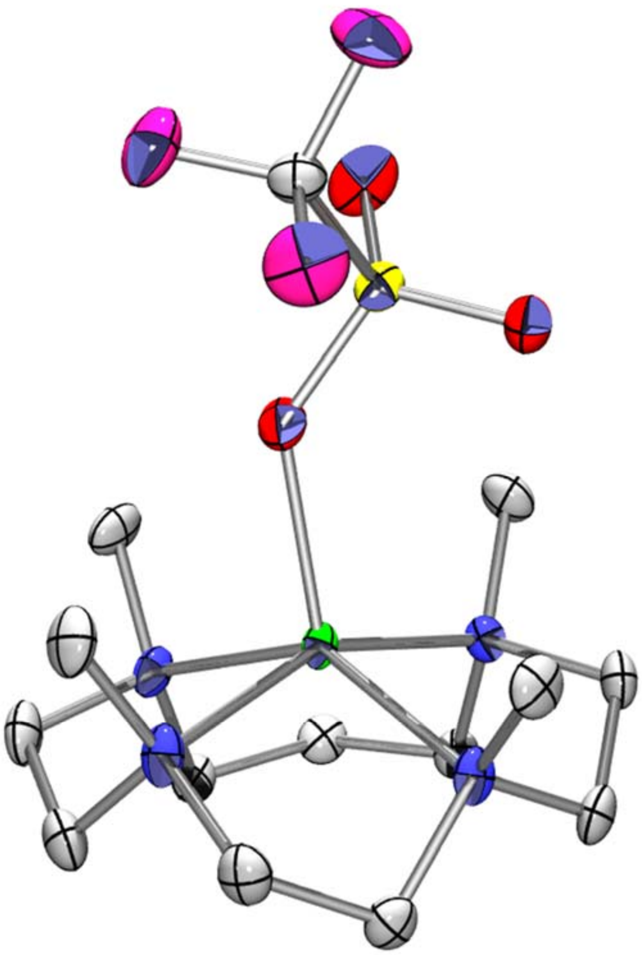

Supplementary Figure 1 | ORTEP-III diagram of $[Co(13\text{-}TMC)(CF_3SO_3)]^+$ in **1** (CCDC-1500945), showing the 50% probability displacement ellipsoids. Hydrogen atoms are omitted for clarity (see also Supplementary Tables 1 – 2 for crystallographic data). Atom colors are aquamarine for Co, blue for N, red for O, gray for C, yellow for S, and pink for F.

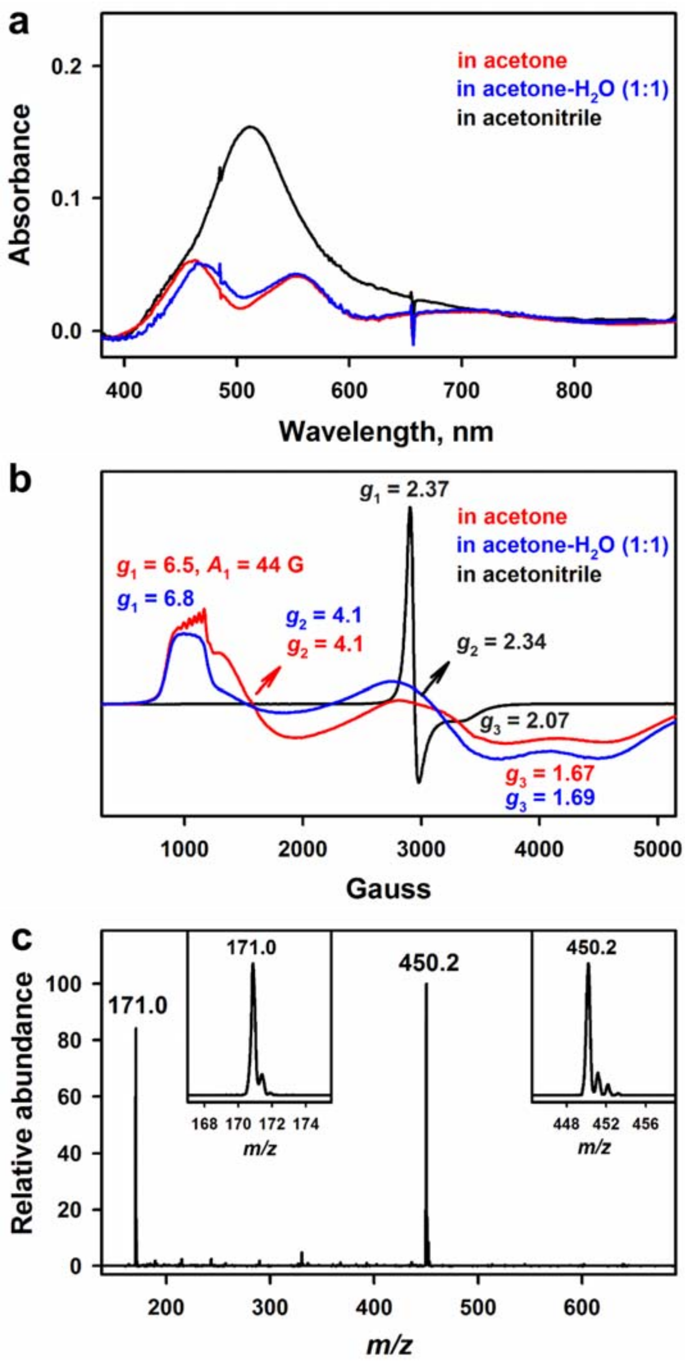

Supplementary Figure 2 | Characterization of Co(13-TMC)(CF_3SO_3)$_2$ (1). a, UV−vis spectra of **1** in acetone (red line), in acetone/H_2O (*v*/*v* = 1/1) (blue line), and in acetonitrile (black line). **b,** X-band EPR spectra of **1** recorded at 5 K in acetone (red line), in acetone/H_2O (*v*/*v* = 1/1) (blue line), and in acetonitrile (black line). **c,** ESI-MS spectrum of **1**. The peaks at *m/z* = 171.0 and 450.2 with isotope distribution patterns (inset) correspond to [(13-TMC)Co^{II}(CH_3CN)]$^{2+}$ (calculated *m/z* = 171.1) and [(13-TMC)Co^{II}(CF_3SO_3)]$^{+}$ (calculated *m/z* = 450.1), respectively.

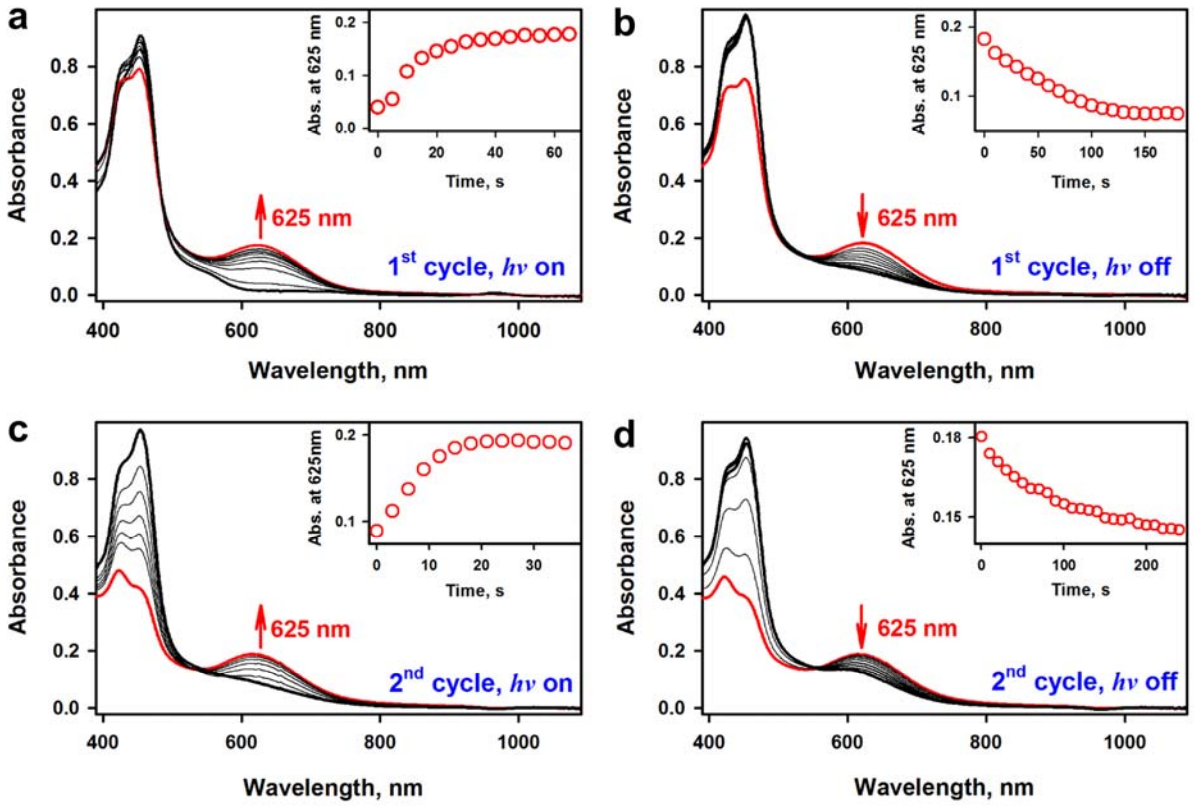

Supplementary Figure 3 | UV−vis spectral changes obtained upon photoirradiation (> 420 nm) of a solution containing **1** (1.0 mM), [Ru^{II}(bpy)$_3$]Cl$_2$ (5.0 × 10^{-2} mM), and $Na_2S_2O_8$ (10 mM) in acetone/H_2O (*v*/*v* = 1/1) at –20 °C [photoirradiation on (**a**) and then off (**b**) for the first cycle, and photoirradiation on again (**c**) and then off (**d**) for the second cycle]. Insets show the time courses monitored at 625 nm for the formation and decay of **2**.

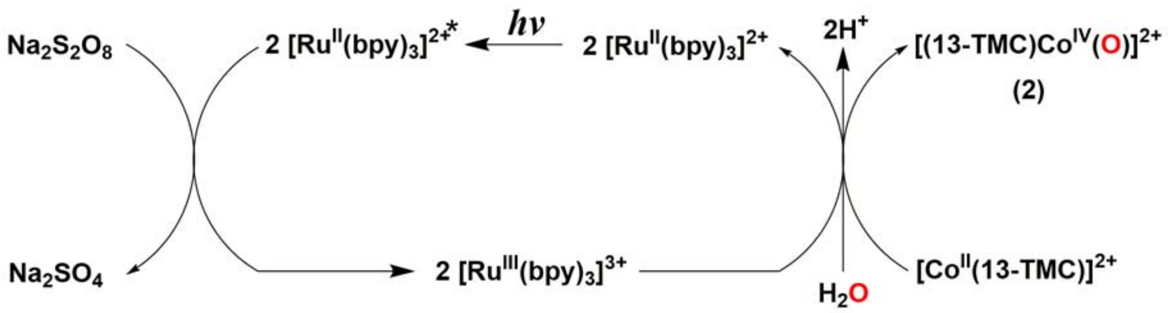

Supplementary Figure 4 | Proposed reaction mechanism of the photocatalytic generation of **2**.

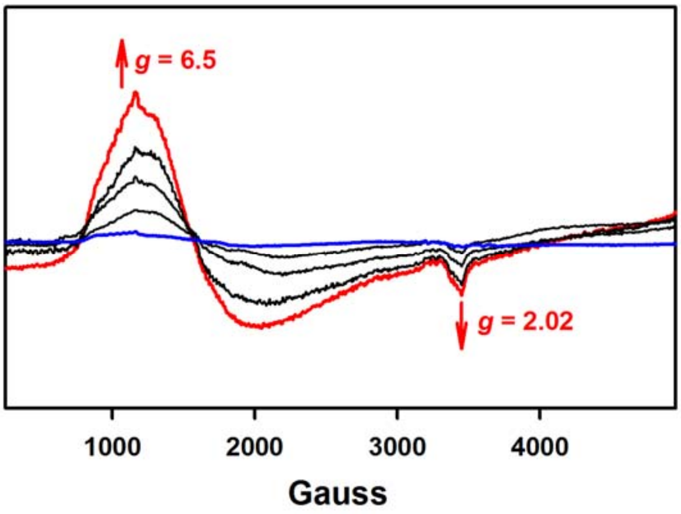

Supplementary Figure 5 | Time-dependent X-band EPR spectral changes observed during the conversion of **3** (blue line) to **2** (red line) upon addition of PhIO (3 equiv.) to a solution of **1** (2.0 mM) in the presence of HOTf (1.2 equiv.) in acetone at –40 °C. The final red line for **2** was generated within 3 min.

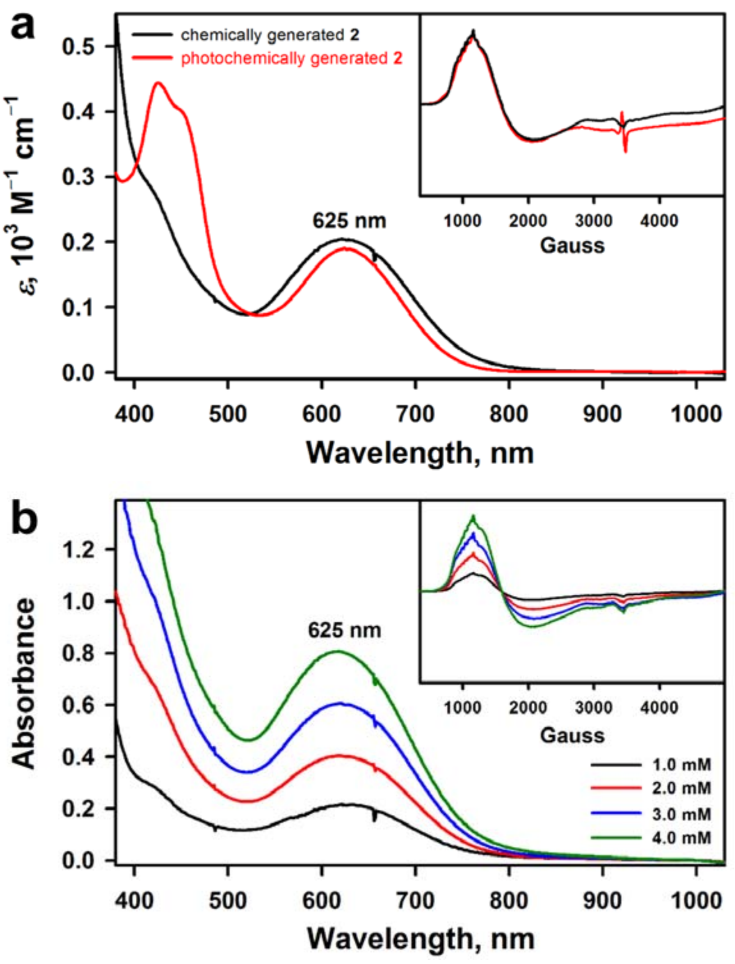

Supplementary Figure 6 | a, Direct comparison of UV−vis spectra of photo- (red line) and chemically (black line) generated **2** (see Fig. 2 for the generation conditions of **2**). Inset shows direct comparison of EPR spectra of photo- (red line) and chemically (black line) generated **2**. **b,** UV−vis spectra of chemically generated **2** with various consentrations [1.0 (balck line), 2.0 (red line), 3.0 (blue line), and 4.0 mM (green line)] in acetone at –40 °C. Inset shows EPR spectra of chemically generated **2** with various consentrations [1.0 (balck line), 2.0 (red line), 3.0 (blue line), and 4.0 mM (green line)] in acetone at –40 °C. All EPR spectra were recorded at 5 K. Notably, based on the observed linear correlation of the intensity of the absorption feature at 625 nm to the intensity of the *S* = 3/2 EPR signal, the 625 nm absorption feature can be considered as a marker band for the presence of a Co^{IV}-O unit.

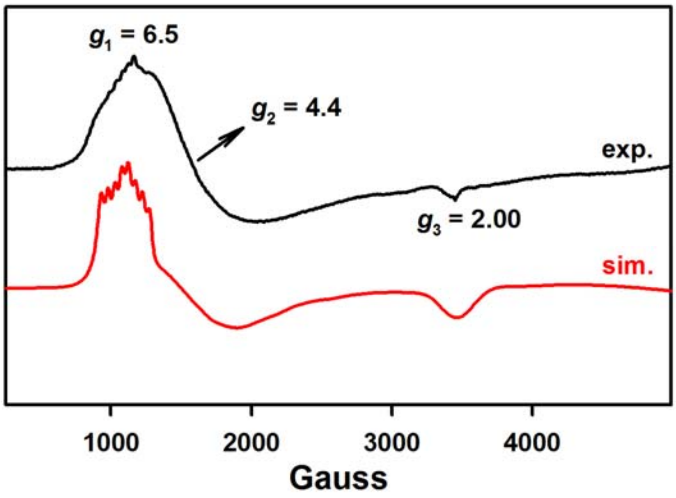

Supplementary Figure 7 | Experimental (black line) and simulated (red line) X-band EPR spectra of 2. Simulation parameters for the S = 3/2 Co^{IV} ground state: D = –1.1 ± 0.4 cm^{-1}, $|E/D|$ = 0.12, g = [2.68, 2.68, 2.085], and A = [50, 66, 0] G.

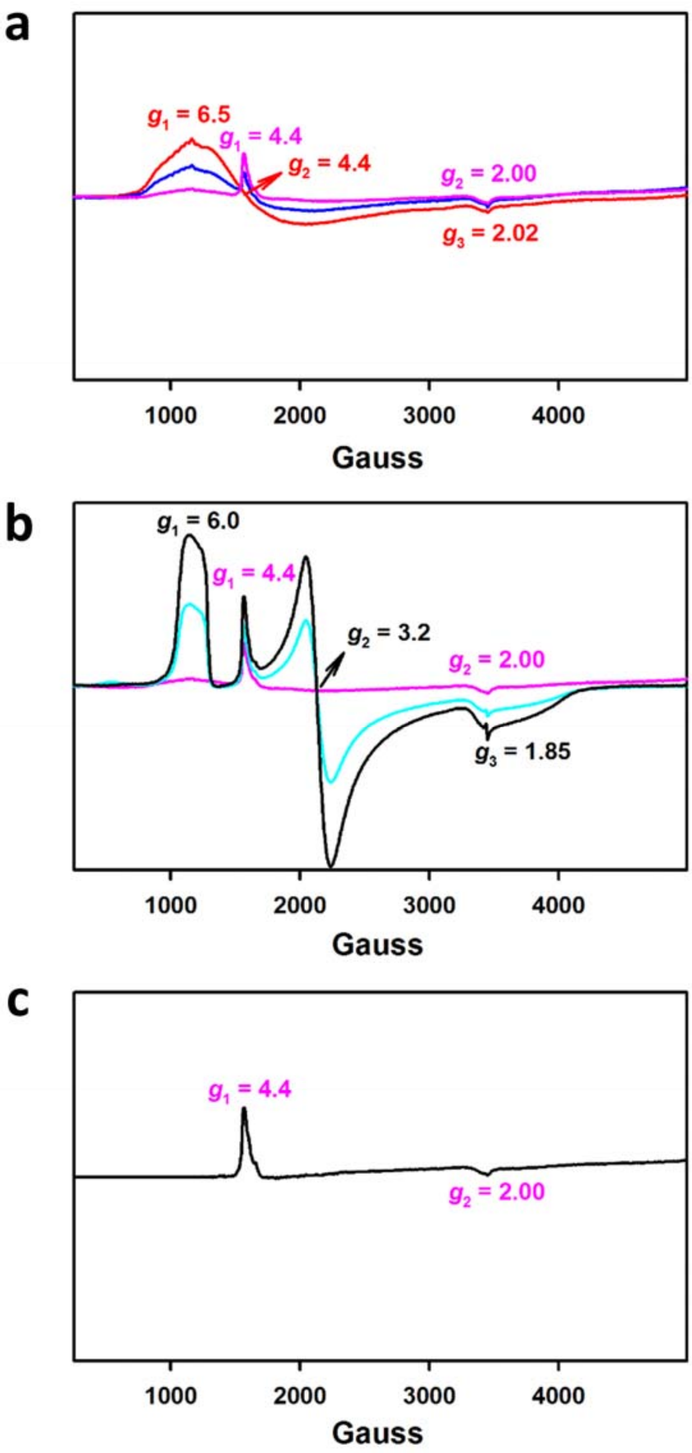

Supplementary Figure 8 | a, X-band EPR spectra of **2** (red line) and **2** plus $Me_{10}Fc$ [0.50 equiv. (blue line) and 1.0 equiv. (pink line)]. **b,** X-band EPR spectra of **2** plus $Me_{10}Fc$ [1.0 equiv. (pink line), 1.5 equiv. (cyan line), and 2.0 equiv. (black line)]. **c,** X-band EPR spectrum of authentic decamethylferrocenium. **2** was generated by reacting Co(13-TMC)$(CF_3SO_3)_2$ (2.0 mM) with PhIO (3.0 equiv.) in the presence of HOTf (1.2 equiv.) in acetone at –40 °C. All spectra were recorded at 5 K.

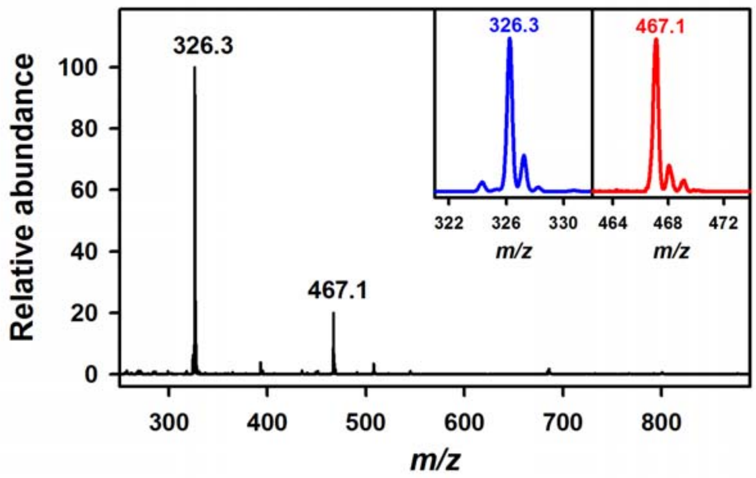

Supplementary Figure 9 | ESI-MS spectrum of a reaction solution of **2** (2.0 mM) plus $Me_{10}Fc$ (1.0 equiv.; 2.0 mM) in the presence of HOTf (1.2 equiv) in acetone at –40 °C. The peaks at *m*/*z* = 326.3 and 467.1 correspond to $Me_{10}Fc^+$ (calculated *m*/*z* = 326.2) and $[(13\text{-TMC})Co^{III}(OH)(OTf)]^+$ (calculated *m*/*z* = 467.1), respectively. Insets show the isotope distribution patterns of the peaks at *m*/*z* = 326.3 (left panel) and 467.1 (right panel). This result is consistent with that of EPR (Supplementary Fig. 7a), demonstrating that **2** was reduced by 1.0 equiv. of $Me_{10}Fc$ to form a $Co^{III}(OH)$ species in the presence of HOTf.

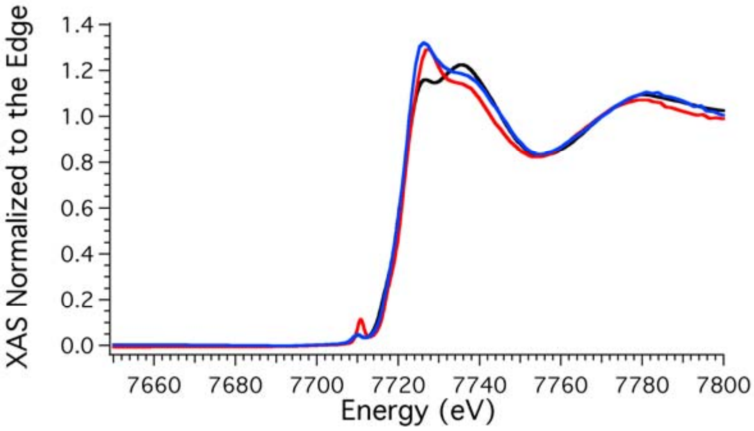

Supplementary Figure 10 | XANES region of the Co K-edge X-ray absorption spectra for $[(14\text{-TMC})Co^{III}(O_2)]^+$ (blue line), $[(12\text{-TMC})Co^{III}(OOH)]^{2+}$ (black line), and $[(13\text{-TMC})Co^{IV}(O)]^{2+}$ (red line).

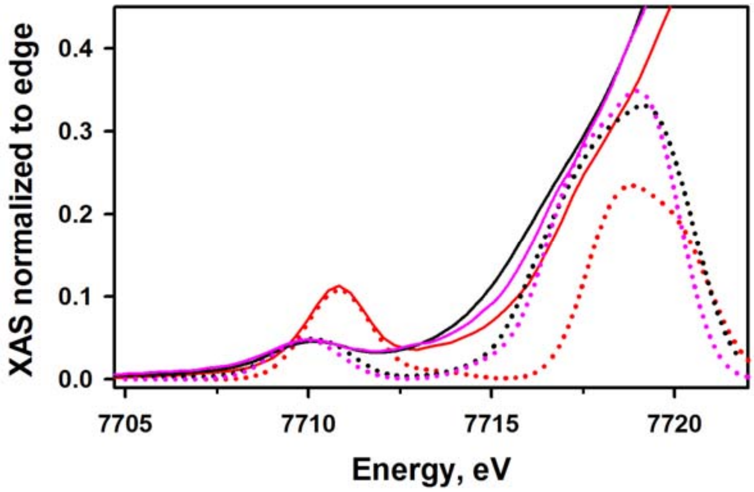

Supplementary Figure 11 | TD-DFT calculated Co K-edge XANES spectrum (PBE0/def2-tzvp(-f)/ZORA) for $[(12\text{-TMC})Co^{III}(OOH)]^{2+}$ (pink), $[(13\text{-TMC})Co^{III}(O_2)]^{+}$ (black) and **2** (red). The experimental data are given as the solid curves and the TD-DFT calculated spectra are given as the dotted curves.

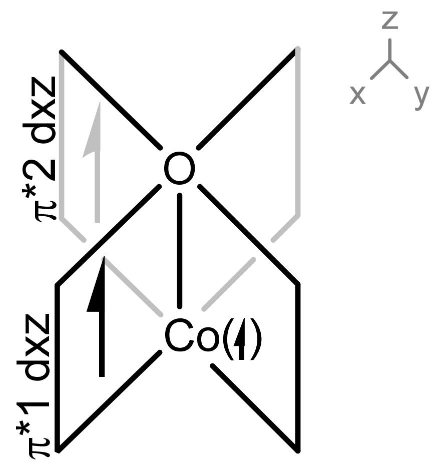

Supplementary Figure 12 | Simplified representation of two of the antibonding orthogonal orbitals constructed from Co-dxz, Co-dyz, O-px and O-py orbitals for the quartet state. The bonding counterparts are NOs 76 and 77. Third SOMO is NO-81 located on Co.

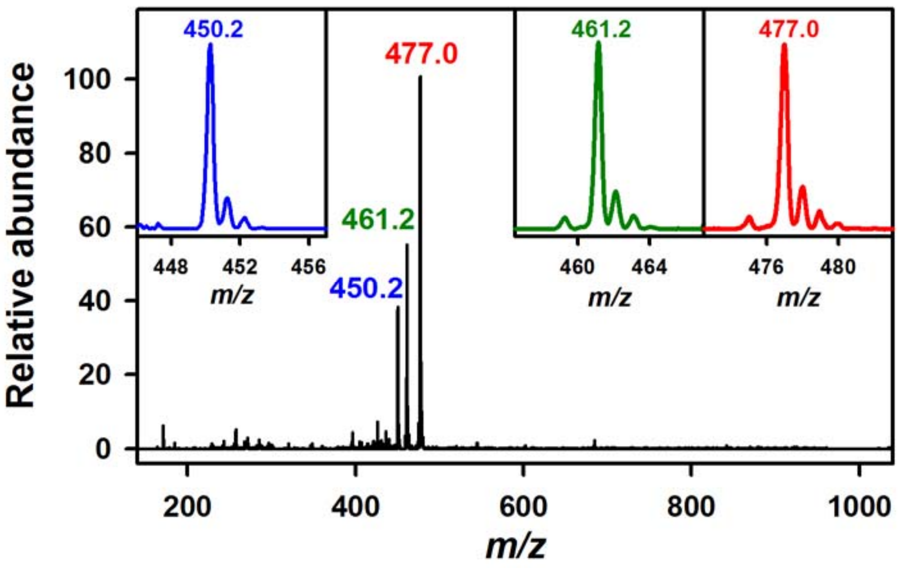

Supplementary Figure 13 | ESI-MS spectrum of the resulting solution obtained after the oxygen atom transfer from **2** (1.0 mM) to $Fe^{II}(14\text{-}TMC)(CF_3SO_3)_2$ (2.0 mM) in the presence of HOTf (1.2 equiv.) in acetone at –40 °C. The peaks at *m*/*z* = 450.2, 461.2, and 477.0 correspond to $[Co^{II}(13\text{-}TMC)(CF_3SO_3)]^+$ (calculated *m*/*z* = 450.1), $[Fe^{II}(14\text{-}TMC)(CF_3SO_3)]^+$ (calculated *m*/*z* = 461.1), and $[Fe^{IV}(O)(14\text{-}TMC)(CF_3SO_3)]^+$ (calculated *m*/*z* = 477.1), respectively. Insets show the isotope distribution patterns of the peaks at *m*/*z* = 450.2 (blue line), 461.2 (green line), and 477.0 (red line). This result is consistent with the UV-vis spectral changes observed in the reaction of **2** with $[Fe^{II}(14\text{-}TMC)]^{2+}$ (see Fig. 4 in text), demonstrating that **2** contains an oxo ligand.

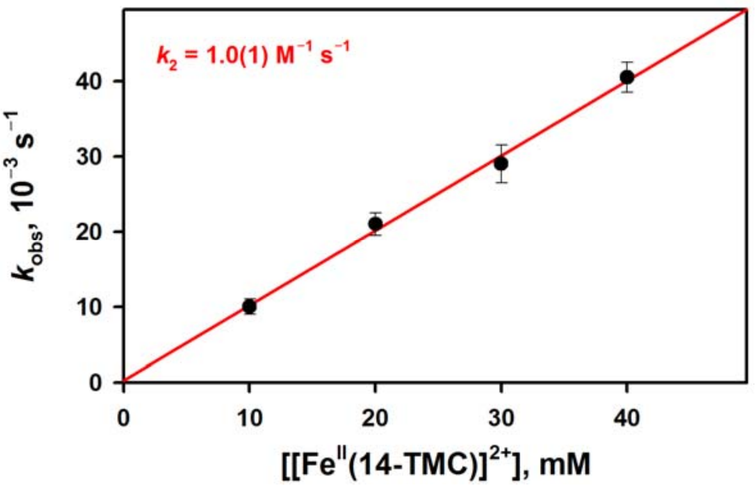

Supplementary Figure 14 | Plot of the pseudo-first-order rate constants (k_{obs}) against the concentrations of $[Fe^{II}(14\text{-TMC})]^{2+}$ complex to determine the second-order rate constants (k_2) in the intermetal OAT from **2** (1.0 mM) to $[Fe^{II}(14\text{-TMC})]^{2+}$ in the presence of HOTf (1.2 equiv.) in acetone at –40 °C.

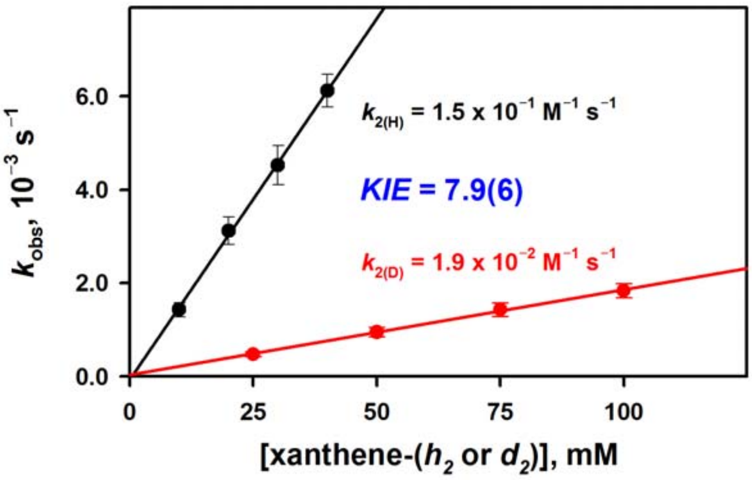

Supplementary Figure 15 | Plots of the pseudo-first-order rate constants (k_{obs}) against the concentrations of xanthene-h_2 (black circles) and xanthene-d_2 (red circles) in the oxidation of xanthene-h_2 and xanthene-d_2 by **2** (1.0 mM) in the presence of HOTf (1.2 equiv.) in acetone at −40 °C to determine the second-order rate constants (k_2) and KIE value.

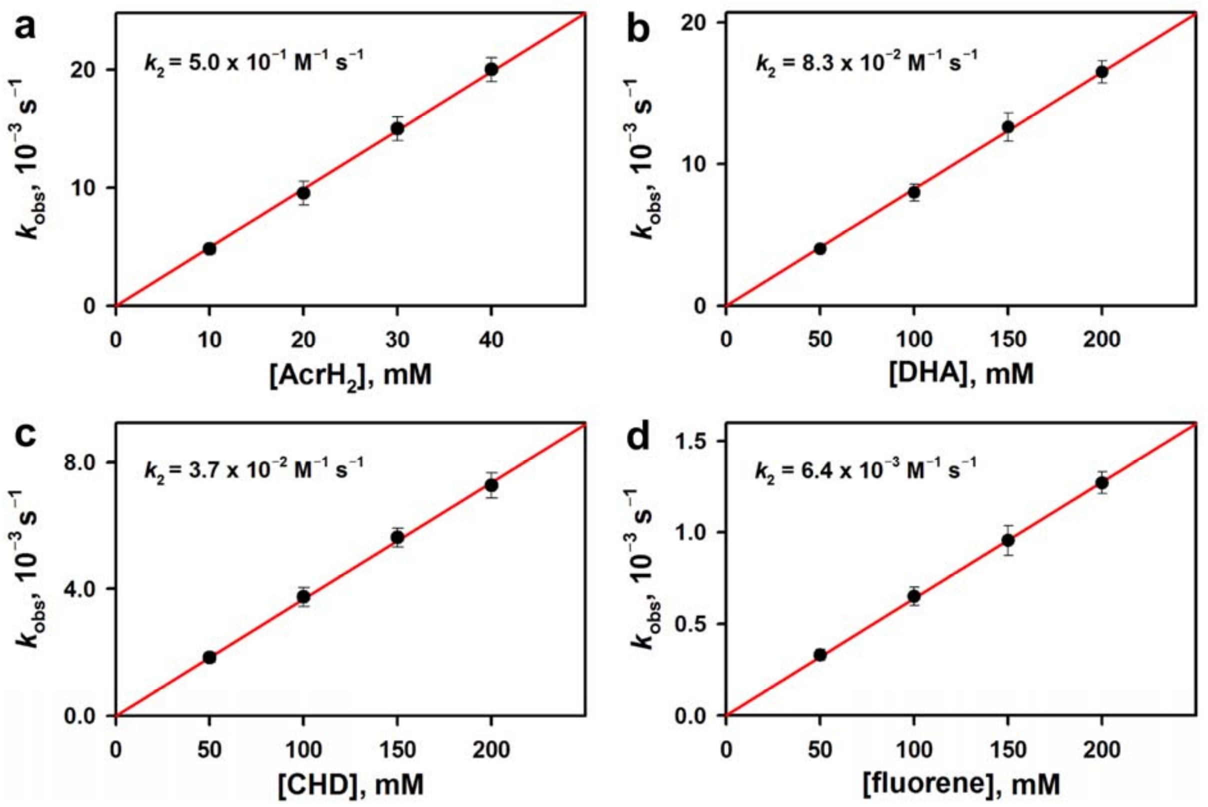

Supplementary Figure 16 | Plots of the pseudo-first-order rate constants (k_{obs}) against the concentrations of hydrocarbons [(**a**) $AcrH_2$, (**b**) 9,10-dihydroanthracene (DHA), (**c**) 1,4-cyclohexadiene (CHD), and (**d**) fluorene] to determine the second-order rate constants (k_2) in the C-H bond activation of hydrocarbons by **2** in the presence of HOTf (1.2 equiv.) in acetone at –40 °C (see also Supplementary Table 12).

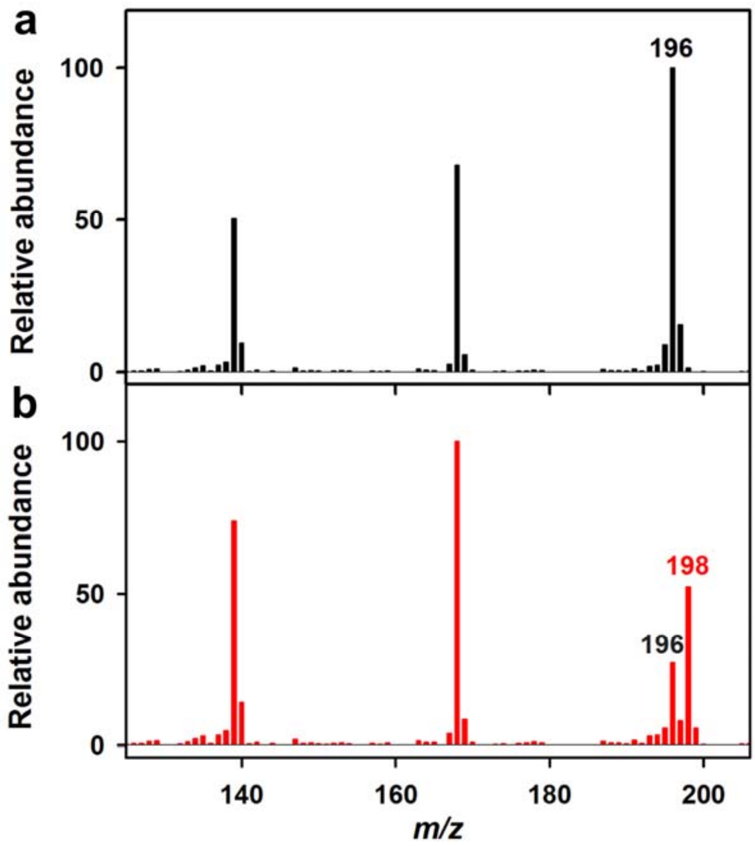

Supplementary Figure 17 | GC-MS spectra of (**a**) xanthone-^{16}O as an authentic sample and (**b**) xanthone-^{18}O produced in the reaction of **2**-^{18}O (1.0 mM, 70(3)% ^{18}O-enriched) and xanthene (20 mM) in the presence of HOTf (1.2 equiv.) under an Ar atmosphere in acetone at –40 °C. The percentage of ^{18}O (67(3)%) in the xanthone product was determined by comparison of the relative abundances at *m/z* = 198 for xanthone-^{18}O and at *m/z* = 196 for xanthone-^{16}O. The ^{18}O-percent of 67(3)% in the xanthone product was almost identical to that in **2**-^{18}O (70(3)%) within the error of experimental measurements.

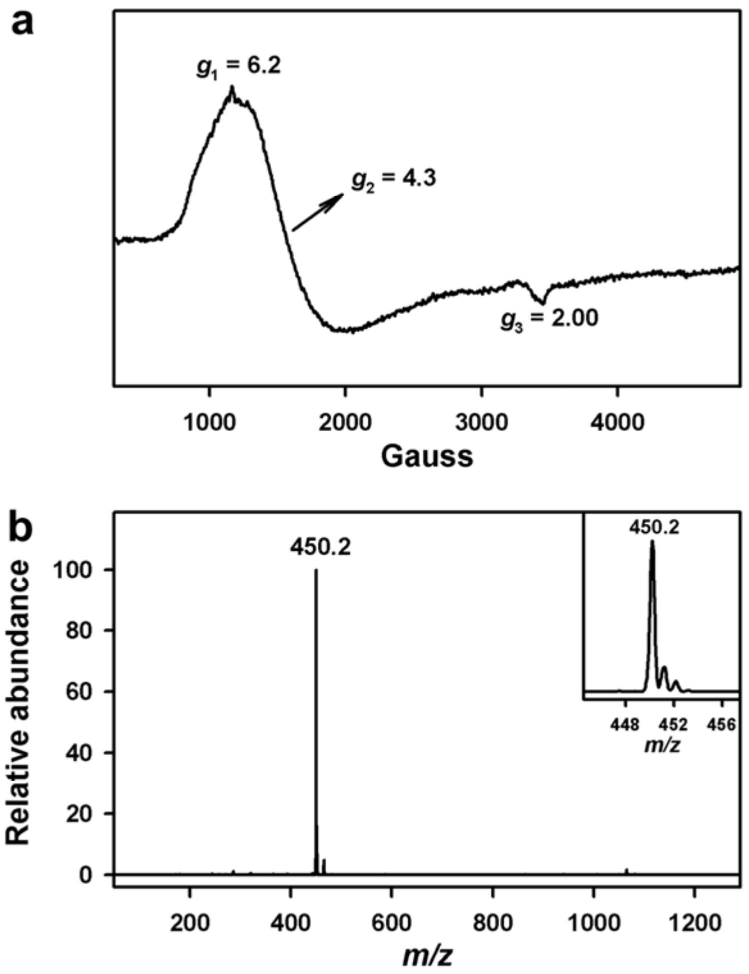

Supplementary Figure 18 | a, X-band EPR spectrum of the final solution obtained after the reaction of **2** (1.0 mM) with xanthene (20 mM) in the presence of HOTf (1.2 equiv) in acetone at –40 °C. The spectrum was recorded at 5 K. **b,** ESI-MS spectrum of the final solution obtained after the reaction of **2** (1.0 mM) with xanthene (20 mM) in the presence of HOTf (1.2 equiv) in acetone at –40 °C. The peak at *m/z* = 450.2 with isotope distribution patterns (inset) corresponds to $[Co^{II}(13\text{-TMC})(CF_3SO_3)]^+$ (calculated *m/z* = 450.1). The results of EPR and ESI MS indicate that a high-spin Co^{II} species was formed as the major product in the reaction of **2** with xanthene.

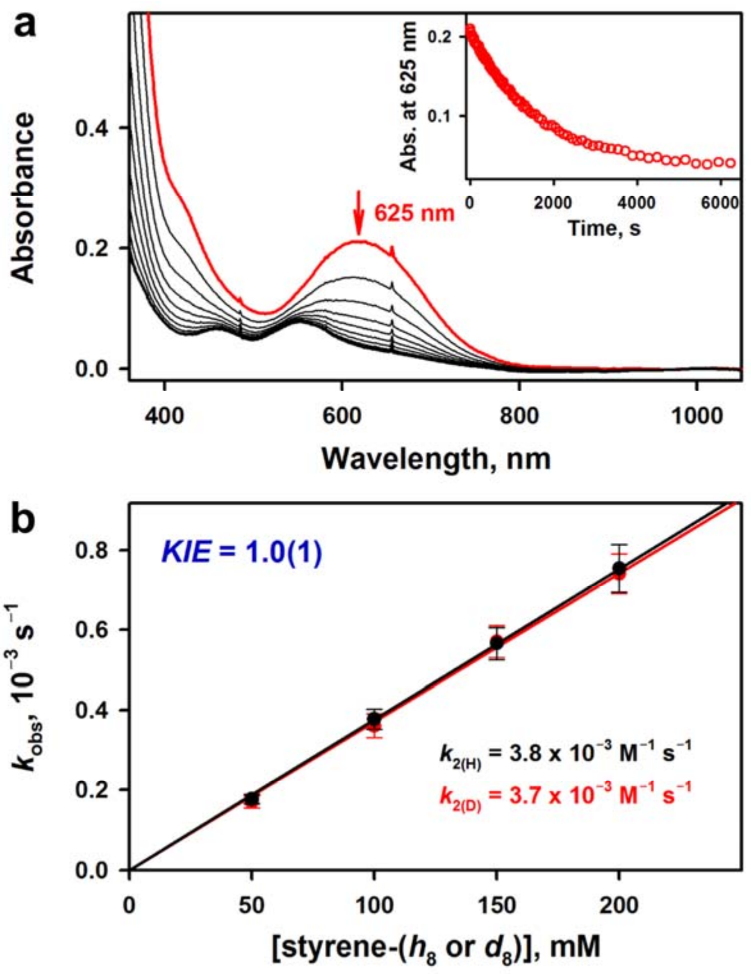

Supplementary Figure 19 | a, UV-vis spectral changes observed in the reaction of **2** (1.0 mM) and styrene (1.5 × 10^2 mM) in the presence of HOTf (1.2 equiv) in acetone at −40 ºC. Inset shows the time course monitored at 625 nm. **b,** Plots of the pseudo-first-order rate constants (k_{obs}) against the concentrations of styrene-h_8 (black circles) and styrene-d_8 (red circles) to determine the second-order rate constants (k_2) for the oxidation of styrene-h_8 and styrene-d_8 by **2** in the presence of HOTf (1.2 equiv) in acetone at −40 ºC.

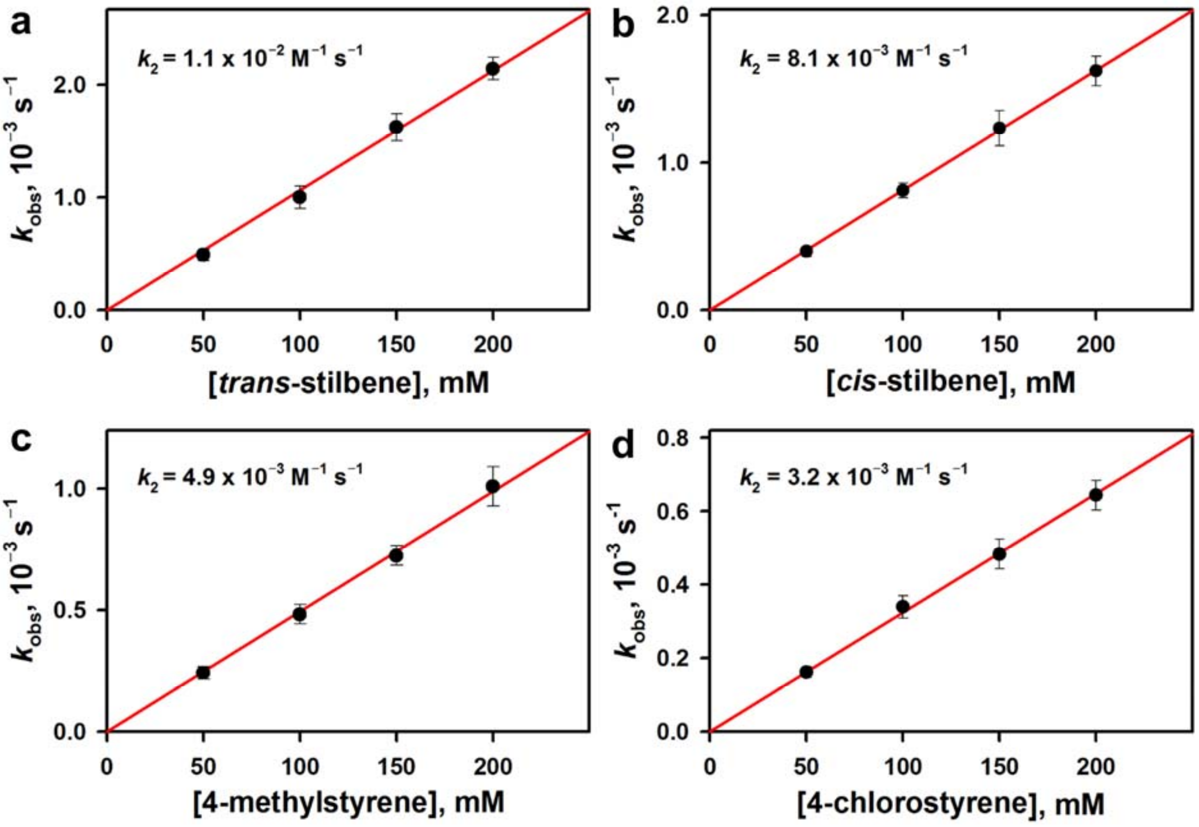

Supplementary Figure 20 | Plots of the pseudo-first-order rate constants (k_{obs}) against the concentrations of various olefins [(**a**) *trans*-stilbene, (**b**) *cis*-stilbene, (**c**) 4-methylstyrene, and (**d**) 4-chlorostyrene] to determine the second-order rate constants (k_2) in the epoxidation of olefins by **2** in the presence of HOTf (1.2 equiv.) in acetone at –40 °C (see also Supplementary Table 14).

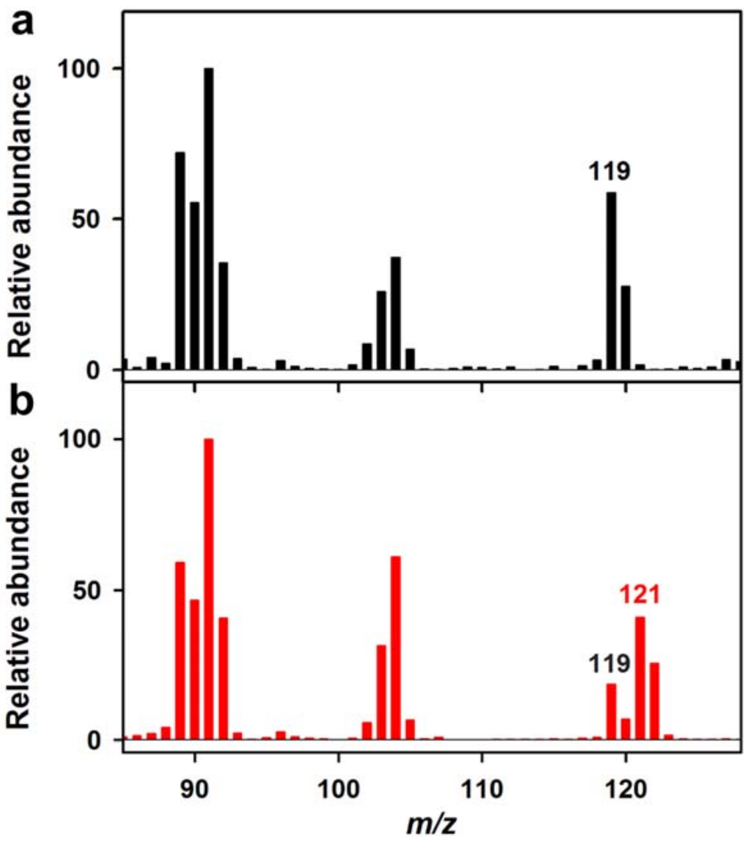

Supplementary Figure 21 | GC-MS spectra of (**a**) styrene oxide-^{16}O as an authentic sample and (**b**) styrene oxide-^{18}O produced in the reaction of **2**-^{18}O (1.0 mM, 70(3)% ^{18}O-enriched) with styrene (100 mM) in the presence of HOTf (1.2 equiv.) under an Ar atmosphere in acetone at –40 °C. The percentage of ^{18}O (68(3)%) in the styrene oxide product was determined by comparison of the relative abundances at *m/z* = 121 for styrene oxide-^{18}O and at *m/z* = 119 for styrene oxide-^{16}O. The ^{18}O-percent of 68(3)% in the styrene oxide product was almost identical to that in **2**-^{18}O (70(3)%) within the error of experimental measurements.

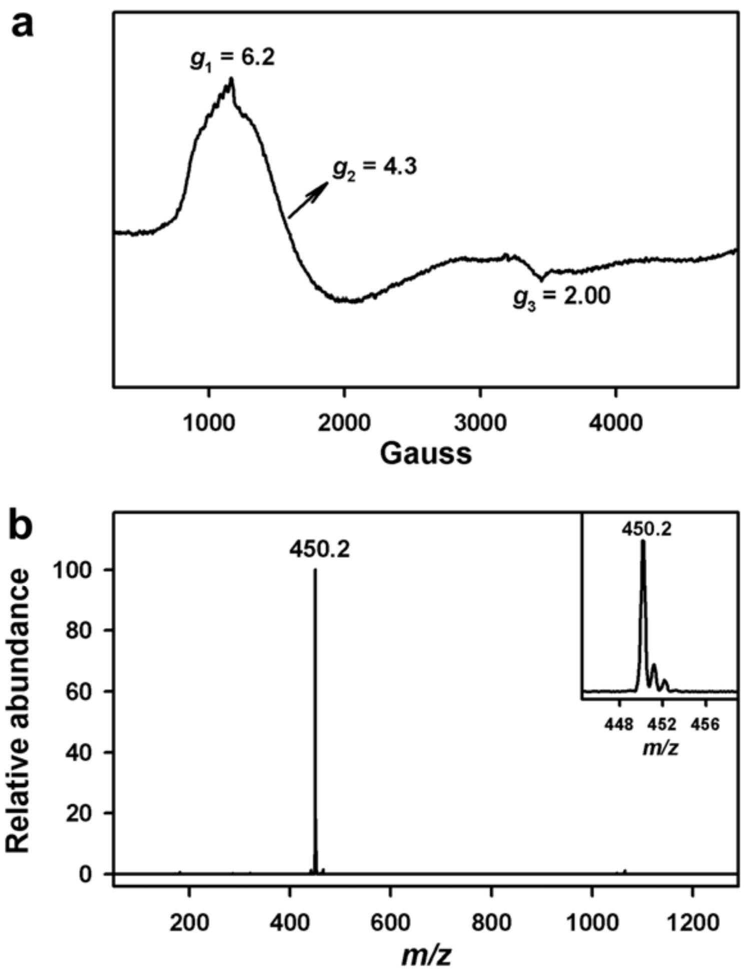

Supplementary Figure 22 | a, X-band EPR spectrum of the solution obtained in the reaction of **2** (1.0 mM) with styrene (100 mM) in the presence of HOTf (1.2 equiv) in acetone at –40 °C. The spectrum was recorded at 5 K. **b,** ESI-MS spectrum of the solution obtained in the reaction of **2** (1.0 mM) and styrene (100 mM) in the presence of HOTf (1.2 equiv) in acetone at –40 °C. The peak at *m/z* = 450.2 with isotope distribution patterns (inset) corresponds to $[Co^{II}(13\text{-}TMC)(CF_3SO_3)]^+$ (calculated *m/z* = 450.1). The results of EPR and ESI-MS indicate that a high-spin Co^{II} species was formed as the major product in the reaction of **2** with styrene.

Supplementary Table 1 | Data collection and structure refinement for **1**.

Empirical formula	$C_{30} H_{60} Co_2 F_{12} N_8 O_{12} S_4$
Formula weight	1198.96
Space group	P 21/n
a, Å	13.0443(2)
b, Å	26.0867(4)
c, Å	14.8477(2)
α, deg	90
β, deg	107.5864(9)
γ, deg	90
V, $Å^3$	4816.28(13)
Z	4
temp, K	100(2)
λ (Mo *Kα*), Å	0.71073
D, g cm^{-3}	1.653
Absorption correction	multi-scan (T_{min} = 0.900, T_{max}= 0.916)
Absorption coefficient (mm^{-1})	0.971
Reflections collected	11866
Independent reflections	9629
Goodness-of-fit on F^2 (S)	1.022
Final R indices [I>2sigma(I)]	R1 = 0.0512, wR2 = 0.1238
R indices (all data)	R1 = 0.0669, wR2 = 0.1340

Supplementary Table 2 | Selected bond distances (Å) and angles (º) for **1**.

Bond Distances (Å)	
Co1-N1	2.118(2)
Co1-N2	2.145(3)
Co1-N3	2.087(3)
Co1-N4	2.175(3)
Co1-O1	2.016(2)
**Bond Angles (º)**	
O1-Co1-N1	100.71(10)
O1-Co1-N2	107.34(11)
O1-Co1-N3	114.04(11)
O1-Co1-N4	95.50(10)
N1-Co1-N2	96.86(10)
N1-Co1-N3	142.99(11)
N1-Co1-N4	83.93(10)
N2-Co1-N3	85.04(11)
N2-Co1-N4	156.51(11)
N3-Co1-N4	80.58(11)

Supplementary Table 3 | Reported model to the EXAFS data for **2** and alternate fits to the EXAFS data.[a,b]

Shell	Final Model	N Only	N and O Only	N, O and C with n unrestrained
N	n = 4<br>r = 2.024(3) Å<br>σ^2 = 0.0046(2) Å^2	n = 2.8(3)<br>r = 2.030(4) Å<br>σ^2 = 0.0026(6) Å^2	n = 4.5(4)<br>r = 2.024(3) Å<br>σ^2 = 0.0055(7) Å^2	n = 4.4(4)<br>r = 2.023(2) Å<br>σ^2 = 0.0053(6) Å^2
O	n = 1<br>r = 1.715(3) Å<br>σ^2 = 0.0028(3) Å^2	-	n = 1.0(2)<br>r = 1.715(6) Å<br>σ^2 = 0.0030(12) Å^2	n = 1.0(2)<br>r = 1.716(3) Å<br>σ^2 = 0.0027(8) Å^2
C	n =4<br>r = 2.676(7) Å<br>σ^2 = 0.0013(9) Å^2	-	-	n = 5(3)<br>r = 2.67(8) Å<br>σ^2 = 0.001(13) Å^2
C	n = 3<br>r = 2.994(7) Å<br>σ^2 = 0.0033(10) Å^2	-	-	n = 8(3)<br>r = 2.996(7) Å<br>σ^2 = 0.010(4) Å^2
C	n = 3<br>r = 3.262(5) Å<br>σ^2 = 0.0018(5) Å^2	-	-	n = 6(2)<br>r = 3.291(11) Å<br>σ^2 = 0.004(2) Å^2
$\varepsilon^{2,c}$	0.61	2.69	2.07	0.57

[a] E_o = 7723.3 eV with the S_0^2 factor set at **1**. [b] The esds in the bond length are based on the refinement to the fit. Actual errors in bond lengths should be taken as ±0.02 Å. [c] Errors are given by $\varepsilon^2 = [n_{idp}/(n_{idp} - n_p)] \times \text{average}[(y_{data} - y_{model})/\sigma^2]$ where n_{idp} is the number of independent data points, n_p is the number of refined parameters, σ is the estimated uncertaininty in the data, y_{data} is the experimental k^3 EXAFS spectrum and y_{model} is the simulated k^3 EXAFS spectrum.

Supplementary Table 4 | Plots and Occupation Numbers (NOON) of CASSCF Natural Orbitals.

#	NO	NOON	Character
82	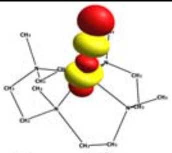	0.263	σ^*–dz^2
81	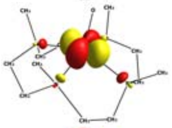	1.004	σ^*–d x^2-y^2
80	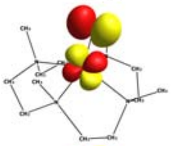	1.089	π^*2–dyz
79	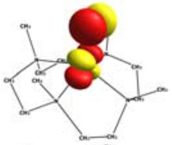	1.095	π^*1–dxz
75	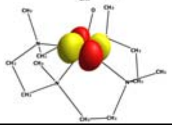	1.979	nb–dxy
77	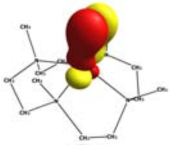	1.886	$\pi2$–dxz
76	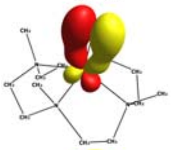	1.894	$\pi1$–dyz
78	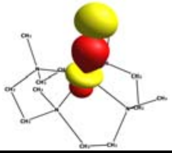	1.759	σ–dz^2

Surfaces are generated at 0.075 au. See Results and Discussion for CASSCF calculations in Supplementary Information for more details.

Supplementary Table 5 | Plots and occupation numbers (NOON) of CASSCF natural orbitals 84 and 83.

#	MO	NOON	Character
84	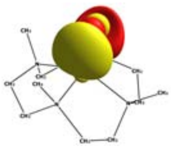	0.156	π*
83	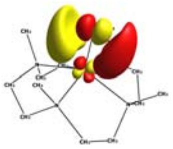	0.165	π*

Surfaces are generated at 0.075 au.

Supplementary Table 6 | Details of the quartet CASSCF wave function.[a]

ALPHA	BETA	Coefficient
1111111000	1111000000	0.874
1110111100	1110000100	0.208
1111111000	1001110000	0.162
1110111100	1101100000	0.150

[a] Configurations with coefficients less than 0.15 are excluded.

Supplementary Table 7 | Bond order analysis for the Co–O bond.

#	Character	NOON	Total		Bond Order
84	π^*	0.156			
83	π^*	0.165	π^*	2.505	π
			π	3.780	0.638
82	σ^*–dz^2	0.263			
81[a]	σ^*–dx^2-dy^2	1.004			
79	π^*1–dxz	1.095			
75[a]	nb-dxy	1.979			
80	π^*2–dyz	1.089	σ^*	0.263	σ
			σ	1.759	0.748
76	$\pi1$–dyz	1.894			
77	$\pi2$–dxz	1.886			
78	σ– dz^2	1.759			

[a] dxy and d x^2-y^2 are nonbonding with respect to the Co–O bond. Lower lying orbitals have a net contribution of zero to the bond order due to generating doubly occupied boding and anti-bonding pairs.

Supplementary Table 8 | Calculated Co–L bond lengths for ^{2}**2**, ^{4}**2**, and ^{6}**2** in Å.

Bond	^{2}**2**	^{4}**2**	^{6}**2**
Co-N1	2.037	2.046	2.218
Co-N2	1.998	2.068	2.160
Co-N3	2.055	2.058	2.176
Co-N4	2.046	2.137	2.160
Co-O	1.709	1.696	1.704

Supplementary Table 9 | Relative electronic energies and Mulliken spin densities and Co-O bond length in ^{2}**2**, ^{4}**2** and ^{6}**2** at the B3LYP/LACVP** level of theory.

State	Mulliken spin density		ΔE, kcal/mol	R(Co-O), Å	<S^2>
	Co	O			
^{2}**2**	-0.040	0.978	4.3	1.709	0.786
^{2}**2-BS**	1.581	-0.481	11.4	1.736	1.818
^{4}**2**	1.658	1.386	**0.0**	1.696	3.821
^{4}**2-oxo**	2.616	-0.240	20.0	1.688	4.368
^{6}**2**	2.706	1.486	12.4	1.704	8.774

Supplementary Table 10 | Relative electronic energies, Mulliken spin densities and Co-O bond length in $^{2}\mathbf{2}$, $^{4}\mathbf{2}$ and $^{6}\mathbf{2}$ at the B3LYP[X] (X= 0, 5, 10, 15 and 20% HF)/LACVP** level of theory.

X(%)	Δ*E*, kcal/mol	Mulliken spin density		R(Co–O), Å
		Co	O	
		$^{4}\mathbf{2}$		
0	0.00	1.639	1.311	1.686
5	0.00	1.652	1.325	1.682
10	0.00	1.658	1.345	1.682
15	0.00	1.651	1.373	1.683
20	0.00	1.653	1.391	1.696
		$^{6}\mathbf{2}$		
0	23.45	2.684	1.378	1.692
5	20.99	2.695	1.402	1.691
10	18.15	2.700	1.426	1.692
15	15.17	2.703	1.452	1.695
20	12.38	2.705	1.490	1.708
		$^{2}\mathbf{2}$		
0	6.16	0.362	0.626	1.679
5	5.93	0.326	0.659	1.676
10	5.67	0.257	0.721	1.678
15	5.19	0.103	0.857	1.692
20	4.66	-0.011	0.952	1.704

Supplementary Table 11 | Relative electronic energies, Mulliken spin densities, Co-O bond length and Raman stretching frequency at the Method/cc-pVTZ(-f) level of theory.

Method	State	Mulliken spin density		ΔE, kcal/mol	r(Co-O), Å	ν, cm^{-1}
		Co	O			
B3LYP-D3	^{4}**2**	1.738	1.330	0.00	1.687	**656**
	^{2}**2**	0.009	0.943	5.69	1.710	678
	^{6}**2**	2.864	1.429	12.27	1.695	718

Supplementary Table 12 | Second-order rate constants, k_2, determined for the C−H bond activation reactions of hydrocarbons by $[Co^{IV}(O)(13\text{-TMC})]^{2+}$ (**2**).[a]

entry	substrate	BDE[b] (kcal mol^{-1})	k_2, M^{-1} s^{-1}	k_2',[c] M^{-1} s^{-1}
1	$AcrH_2$	73.7	$5.0(4) \times 10^{-1}$	$2.5(2) \times 10^{-1}$
2	xanthene	75.5	$1.5(1) \times 10^{-1}$	$7.5(6) \times 10^{-2}$
3	xanthene-d_2	-	$1.9(2) \times 10^{-2}$	$9.5(8) \times 10^{-3}$
4	9,10-dihydroanthracene	77.0	$8.3(7) \times 10^{-2}$	$2.1(2) \times 10^{-2}$
5	1,4-cyclohexadiene	78.0	$3.7(3) \times 10^{-2}$	$9.2(8) \times 10^{-3}$
6	fluorene	80.0	$6.4(6) \times 10^{-3}$	$3.2(3) \times 10^{-3}$

[a] Reactions of **2** (1.0 mM) with various substrates were carried out in the presence of HOTf (1.2 equiv.) in acetone at –40 °C. [b] Taken from reference 59 in the text. [c] All k_2 values in C–H bond activation reactions were adjusted for reaction stoichiometry to yield k_2' values based on the number of equivalent target C–H bonds in the substrates (e.g., two for xanthene and four for 9,10-dihydroanthracene).

Supplementary Table 13 | Product analysis for the reactions of **2** with xanthene, 9,10-dihydroanthracene, 1,4-cyclohexadiene, and fluorene in the presence of HOTf (1.2 equiv.) under an argon atmosphere in acetone at –40 °C.

entry	substrate	product	yield (%)
1	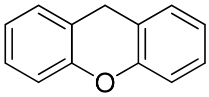<br>xanthene	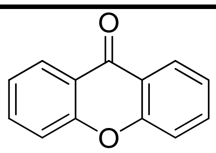<br>xanthone	45(3)
2	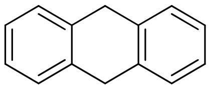<br>9,10-dihydroanthracene	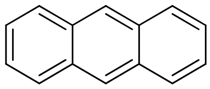<br>anthracene	88(5)
3	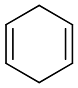<br>1,4-cyclohexadiene	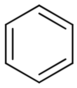<br>benzene	98(2)
4	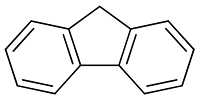<br>fluorene	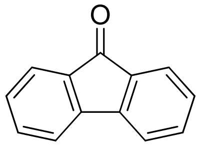<br>9-fluorenone	44(3)

Supplementary Table 14 | Oxidation potentials (E_{ox}) of various olefins and the second-order rate constants determined for the epoxidation of olefins by **2**.[a]

substrate	E_{ox}, V *vs* SCE[b]	k_2, M^{-1} s^{-1}	log k_2
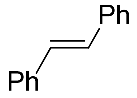 *trans*-stilbene	1.44	1.1×10^{-2}	−1.96
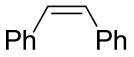 *cis*-stilbene	1.55	8.1×10^{-3}	−2.09
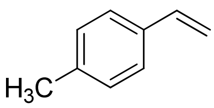 4-methylstyrene	1.73	4.9×10^{-3}	−2.31
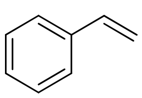 styrene	1.94	3.8×10^{-3}	−2.42
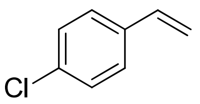 4-chlorostyrene	1.97	3.2×10^{-3}	−2.49

[a] Reactions with **2** (1.0 mM) were carried out in the presence of HOTf (1.2 equiv) in acetone at –40 °C. [b] One-electron oxidation potentials were determined by the second-harmonic alternating current voltammetry (SHACV) measurements at scan rate of 4 mV s^{-1} using Pt working electrode.

Supplementary Table 15 | Product analyses for the reactions of **2** with various olefins in the presence of HOTf (1.2 equiv) under an argon atmosphere in acetone at –40 °C.

entry	substrate	product	yield (%)
1	*trans*-stilbene	*trans*-stilbene oxide	87(5)
		cis-stilbene oxide	n.d.[a]
2	*cis*-stilbene	*cis*-stilbene oxide	76(4)
		trans-stilbene oxide	2.3(2)
3	4-methylstyrene	4-methylstyrene oxide	74(5)
		4-chlorophenylacetaldehyde	2.3(3)
4	styrene	styrene oxide	77(5)
		2-phenylacetaldehyde	1.3(2)
5	4-chlorostyrene	4-chlorostyrene oxide	75(4)
		4-chlorophenylacetaldehyde	1.6(3)

[a] n.d. = not detected.

Supplementary Note 1

Results and Discussion for CASSCF calculations. Natural orbitals and the corresponding occupancies suggest that the Co–O bond order is 1.39 due to a total of 0.75 σ and 0.64 π bonds (Supplementary Table 7). Except the bonding and antibonding orbitals paired as NO-78 and NO-82, all other orbitals are essentially doubly or singly occupied. The three SOMOs are NO-79, NO-80, and NO-81 and they host the unpaired α electrons of the quartet state. While NO-81, being the $d_{x^2-y^2}$ orbital, is clearly confined to cobalt d-space, the remaining two are combinations of Co-d and oxygen-p atomic orbitals. Notably, NO-79 and NO-80 are not linearly dependent and are mutually orthogonal. Thus, although one might think of the two orbitals as two mixed cobalt/oxygen-hybrids and might prefer to assign a single electron to the two centers by renormalizing these two orbitals for simplicity, such an assignment is misleading. Consequently, instead of using |Co(d)(↑)⟩ |Co(d)(↑)⟩ |O(p)(↑)⟩ configuration for the quartet state, a more realistic description is |Co(d)(↑)⟩ Co(d)±O(p)(↑)⟩ Co(d)±O(p)(↑)⟩ where the latter two cobalt/oxygen-hybrids are orthogonal as schematized in Supplementary Fig. 12. Hence the oxygen is not a conventional and well localized radical center yet there is significant α-electron density along the Co–O bond and around oxygen. Such an electronic structure also explains the short Co–O bond length due to an involvement of NOs 79 and 80 in enhanced exchange along the Co–O bond.

Supplementary Note 2

Results and Discussion for DFT Calculations. The high-spin, intermediate and low-spin configurations of the Co(IV)-d^5 centered within the approximately square-pyramidal coordination geometry were considered to afford the doublet, quartet and sextet complexes ^{2}**2**, ^{4}**2** and ^{6}**2**, respectively.

The lowest energy configuration according to our calculations is the intermediate-spin state ^{4}**2** (Supplementary Table 9). The low spin-state ^{2}**2** is slightly higher in energy (+4.3 kcal/mol), while the high-spin state ^{6}**2** is significantly less stable (+11.4 kcal/mol) than ^{4}**2**.

^{2}**2**, exhibits an oxyl radical character on oxygen with a Mulliken spin density of 0.98α on the O atom (Supplementary Table 9). ^{4}**2** with a Mulliken spin density of 1.39α on the O atom indicates a significant oxene diradical character on oxygen. The same holds for ^{6}**2** with a

Mulliken spin density of 1.49α on the O atom. The NBO bond order is calculated to be 0.88 for **^{2}2**, pointing to a single bond character in the Co–O bond. Interestingly, for **^{4}2** the NBO bond-order is 1.39, higher than expected for the resonance form Co(II)-oxene, suggesting a significant double bond character in the Co–O bond. (The electronic structure of **^{4}2** is discussed in more detail below in the CASSCF section). These bond order values are in line with the shorter Co–O bond length (1.696 Å) in **^{4}2**, when compared to **^{2}2** (1.709 Å).

We were able to capture and calculate the **^{4}2**-oxo state with basically zero (-0.24*α*) spin density on the O atom, that can be described as $[Co^{IV}{=}O]^{2+}$ with a strong oxo character on oxygen. **^{4}2-oxo** is 20.0 kcal/mol higher in energy than **^{4}2**. Additional doublet state **^{2}2-BS** was obtained from **^{4}2**, by flipping spin on O so that spins on Co and O in the new state are antiferromagnetically coupled. Surprisingly, **^{2}2-BS** is also higher in energy than **^{4}2** by 11.4 kcal/mol.

We examined the effect of the amount of exact HF exchange on the relative stability of states, Mulliken spin distribution and geometrical parameters (cf. Supplementary Table 10). The increase of the amount of HF exchange, i.e. X(%) results in a smaller energy gap between the states. As well, the increase of X(%) expectedly leads to a higher localization of Mulliken spin density on Co and O atoms, which in turn, results in a longer Co-O bond length. Despite of these quantitative differences, all B3LYP[X] functionals consistently point to the same qualitative picture: **^{4}2** is the most stable state; **^{4}2** and **^{6}2** exhibit a significant oxene character on oxygen, while **^{2}2** – oxyl character on oxygen.

The results of high quality DFT calculations employing B3LYP-D3 and M06 functional in conjunction with the cc-pVTZ(-f) basis set are shown in Supplementary Table 11. B3LYP-D3 results are not very different from those by B3LYP and a smaller basis set. M06, on the other hand, predicts a different relative stability of states: **^{4}2** is still the lowest energy configuration, but now it is closely followed by **^{6}2** (+1.5 kcal/mol), while **^{2}2** has become the least stable one among three (+19.7 kcal/mol). In terms of Mulliken spin density distribution both methods yield similar results to those by B3LYP/LACVP**.

Both methods, B3LYP-D3 (656 cm^{-1}) and M06 (693 cm^{-1}) underestimate the Co-O Raman stretching frequency, though M06 is in a better agreement with the experimental value of 770 cm^{-1} (cf. Supplementary Table 11).

Supplementary Methods

Materials. All chemicals were purchased from Aldrich and TCI with the maximum purity available, and used as received unless otherwise indicated. Solvents for air- and moisture-sensitive manipulations were dried and deoxygenated under an argon atmosphere prior to use.[1] All air- and moisture-sensitive manipulations were carried out using standard Schlenk line techniques or in a drybox with an argon atmosphere. $H_2{}^{18}O$ (95% ^{18}O-enriched) was purchased from ICON Services Inc. (Summit, NJ, USA). 10-methylacridinium iodide ($AcrH^+I^-$) was synthesized as described in the literature.[2] 9,10-Dihydro-10-methylacridine ($AcrH_2$) was prepared by reducing 10-methylacridinium iodide ($AcrH^+I^-$) with $NaBH_4$ in methanol and purified by recrystallization from ethanol.[3-4] The deuterated xanthene (xanthene-d_2) was prepared according to the reported method.[5] $Co(CF_3SO_3)_2$ was synthesized from cobalt powder and trifluoromethanesulfonic acid (CF_3SO_3H) by modifying a published procedure,[6] and recrystallized from acetonitrile/ether. Iodosylbenzene (PhIO) was prepared according to the reported methods.[7] Purity of the iodosylbenzene was determined by iodometric titration.

DFT Calculations. All DFT[8-9] calculations were carried out using Jaguar 8.9 suite[10] of ab initio quantum chemistry programs. Geometry optimizations were performed with B3LYP[11-15] functional and the 6-31G** basis set.[16] Co was represented using the Los Alamos LACVP basis that includes effective core potential.[17-18] Selected geometries were reoptimized using B3LYP-D3[19-20] and M06[21] functionals in conjunction with the Dunning's correlation consistent triple-ζ basis set cc-pVTZ(-f)[22] that includes a double set of polarization functions. For Co, a modified version of LACVP, designated as LACV3P, in which the exponents were decontracted to match the effective core potential with triple-ζ quality was used. Analytical vibrational frequencies within the harmonic approximation were computed with the corresponding basis sets to confirm proper convergence to well-defined minima or saddle points on the potential energy surface.

The functional dependence on exact HF exchange was tested using modified forms of B3LYP by varying the parameter "a" in Equation 1. Parameters b and c were kept fixed at 0.72 and 0.81 respectively. The resultant functionals were referred to as B3LYP[X], where "X" is the value of "a" expressed as a percentage.

$$\mathrm{B3LYP} = (1-a)E_x^{LDA} + aE_x^{HF} + b\Delta E_x^{B} + (1-c)E_c^{LDA} + cE_c^{LYP} \qquad (1)$$

Multireference Calculations. Complete active space self-consistent field (CASSCF)[23] calculations were performed with GAMESS-US (Dec. 2014) suite of programs.[24] The active space is selected from the frontier orbitals of an unrestricted SCF calculation and is comprised of five Co d-orbitals, three oxygen p-orbitals and two virtual Co d-orbitals. Overall this constitutes an eleven electrons in ten orbitals active space, i.e. CAS(11,10). The most important active orbitals are discussed in the main text, see the two virtual Co d-based natural orbitals in Supplementary Table 5. The quartet state was computed to have significant multi-reference character. See Supplementary Table 6 for the details of the wave function.

Supplementary References

1. Armarego, W. L. F. & Chai, C. L. L. *Purification of Laboratory Chemicals,* 6th ed.; Pergamon Press: Oxford, 2009.
2. Joseph, J. et al. Tuning of intercalation and electron-transfer processes between DNA and acridinium derivatives through steric effects. *Bioconjugate Chem.* **15**, 1230–1235 (2004).
3. Fukuzumi, S. *et al.* Energetic comparison between photoinduced electron-transfer reactions from NADH model compounds to organic and inorganic oxidants and hydride-transfer reactions from NADH model compounds to p-benzoquinone derivatives. *J. Am. Chem. Soc.* **109**, 305–316 (1987).
4. Fukuzumi, S. *et al.* Electron-transfer oxidation of 9-substituted 10-methyl-9,10-dihydroacridines. Cleavage of the carbon-hydrogen vs. carbon-carbon bond of the radical cations. *J. Am. Chem. Soc.* **115**, 8960–8968 (1993).
5. Company, A. *et al.* Modeling the *cis*-oxo-labile binding site motif of non-heme iron oxygenases: Water exchange and oxidation reactivity of a non-heme iron(IV)-oxo compound bearing a tripodal tetradentate ligand. *Chem. Eur. J.* **17**, 1622–1634 (2011).
6. Inada, Y. *et al.* Structural characterization and formation mechanism of sitting-atop (SAT) complexes of 5,10,15,20-tetraphenylporphyrin with divalent metal ions. Structure of the Cu(II)−SAT complex as determined by fluorescent extended X-ray absorption fine structure. *Inorg. Chem.* **39**, 4793–4801 (2000).
7. Saltzman, H. & Sharefkin, J. G. Iodosobenzene. *Org. Synth.* **43**, 60–61 (1963).
8. Parr, R. G. & Yang, W. *Density Functional Theory of Atoms and Molecules*; Oxford University Press, New York, 1989.
9. Ziegler, T. Approximate density functional theory as a practical tool in molecular energetics and dynamics. *Chem. Rev.* **91**, 651–667 (1991).
10. Jaguar version 8.9, Schrödinger, LLC, New York, NY, (2015).
11. Becke, A. D. Density-functional exchange-energy approximation with correct asymptotic behavior. *Phys. Rev. A.* **38**, 3098–3100 (1988).
12. Becke, A. D. Density-functional thermochemistry. III. The role of exact exchange. *J. Chem. Phys.* **98**, 5648–5652 (1993).
13. Lee, C. *et al.* Development of the Colle-Salvetti correlation-energy formula into a functional of the electron density. *Phys. Rev. B.* **37**, 785–789 (1988).

14. Slater, J. C. *Quantum Theory of Molecules and Solids*, Vol. 4: *The Self-Consistent Field for Molecules and Solids*, McGraw-Hill, New York, 1974.
15. Vosko, S. H. *et al*. Accurate spin-dependent electron liquid correlation energies for local spin density calculations: a critical analysis. *Can. J. Phys.* **58**, 1200–1211 (1980).
16. Hariharan, P. C. & Pople, J. A. The influence of polarization functions on molecular orbital hydrogenation energies. *Theor. Chim. Acta* **28**, 213–222 (1973).
17. Hay, P. J. & Wadt, W. R. Ab initio effective core potentials for molecular calculations. Potentials for the transition metal atoms Sc to Hg. *J. Chem. Phys.* **82**, 270–282 (1985).
18. Hay, P. J. & Wadt, W. R. Ab initio effective core potentials for molecular calculations. Potentials for K to Au including the outermost core orbitals. *J. Chem. Phys.* **82**, 299–310 (1985).
19. Grimme, S. *et al.* A consistent and accurate ab initio parametrization of density functional dispersion correction (DFT-D) for the 94 elements H-Pu. *J. Chem. Phys.* **132**, 154104–154119 (2010).
20. Goerigk, L. & Grimme, S. A thorough benchmark of density functional methods for general main group thermochemistry, kinetics, and noncovalent interactions. *Phys. Chem. Chem. Phys.* **13**, 6670–6688 (2011).
21. Zhao, Y. & Truhlar, D. G. The M06 suite of density functionals for main group thermochemistry, thermochemical kinetics, noncovalent interactions, excited states, and transition elements: two new functionals and systematic testing of four M06-class functionals and 12 other functionals. *Theor. Chem. Acc.* **120**, 215–241 (2008).
22. Dunning, T. H., Jr. Gaussian basis sets for use in correlated molecular calculations. I. The atoms boron through neon and hydrogen. *J. Chem. Phys.* **90**, 1007–1023 (1989).
23. Schmidt, M. W. & Gordon, M. S. The Construction and Interpretation of MCSCF Wavefunctions. *Annu. Rev. Phys. Chem.* **49**, 233–266 (2003).
24. Schmidt, M. W. *et al.* General atomic and molecular electronic structure system. *J. Comput. Chem.* **14**, 1347–1363 (1993).

B3LYP/cc-pVTZ(-f) Cartesian coordinates

^{2}2

Co -0.010952444 -0.109761439 -0.222569276
C 1.971730237 1.986692163 -0.979468992
N 1.327621519 0.736605797 -1.481038719
C 0.640010923 1.012058290 -2.791511691
C -0.696017831 0.296316960 -2.813617731
N -1.391488893 0.504349098 -1.506608504
C -2.546725623 -0.428719581 -1.342085131
C -2.773552700 -0.639813697 0.133361401
N -1.495959996 -1.061841941 0.807882362
C -1.643428166 -0.705610346 2.250245148
C 2.320499763 -0.368184672 -1.601902073
C 2.749789138 -0.787059275 -0.215209988
N 1.541618816 -1.094170860 0.638048482
C 1.862331920 -0.719724912 2.045401282
C -1.824755792 1.919659132 -1.324273218
C 1.253703741 -2.566213214 0.513124098
C 0.004013212 -3.067133856 1.216842524
C -1.302626539 -2.538382054 0.647379430
H -3.437487903 -0.025233409 -1.824025652
H -2.303338406 -1.363007064 -1.841995481
H -3.093776806 0.283361797 0.604227881
H -3.552457378 -1.381982676 0.310017198
H -2.144431175 -3.046182202 1.122271041
H -1.351824297 -2.768183018 -0.418197885
H 0.064072042 -2.910236356 2.290498847
H -0.009946257 -4.150400872 1.089142040
H 2.124888828 -3.103303483 0.893254939
H 1.184452150 -2.790959558 -0.553466710
H 3.308834446 0.006000884 0.266857312
H 3.405423523 -1.655694520 -0.257064756
H 3.180508954 -0.051527544 -2.195149372
H 1.846918267 -1.193856176 -2.134837640
H 0.509933003 2.083742326 -2.889561610
H 1.263650398 0.689810539 -3.624142163
H -1.318413089 0.635363530 -3.643436397
H -0.551859051 -0.778138796 -2.938794084
H -2.299209326 2.029463398 -0.357063004
H -0.966001133 2.577622208 -1.340993394
H -2.519206216 2.195818998 -2.117017708
H -0.812088584 -1.075645762 2.831244662
H -1.678130956 0.372652108 2.341654824
H -2.560922902 -1.149176563 2.637856599
H 1.072675102 -1.027819287 2.714424763
H 2.791030077 -1.200012940 2.353608347
H 1.960771834 0.359314013 2.105244279
H 2.733941797 2.310019998 -1.688391217
H 1.218871886 2.756436302 -0.864740533
H 2.410991473 1.825991287 -0.004850099
O -0.046196232 1.242570721 0.824012131

^{4}2

Co -0.021081334 0.010661011 -0.128634483
C 2.023281916 1.969796236 -0.958873560
N 1.321327319 0.751941753 -1.460209959
C 0.627338635 1.063092659 -2.763526804
C -0.660317439 0.271522245 -2.859080918
N -1.398929577 0.428591342 -1.576361136
C -2.515325208 -0.540160370 -1.408843355
C -2.777215879 -0.687555776 0.072216785
N -1.506665716 -1.047155149 0.802059220
C -1.688006918 -0.678271620 2.237176866
C 2.266396555 -0.391420861 -1.650732227
C 2.742060278 -0.889225344 -0.311074776
N 1.562137247 -1.188509439 0.574056777
C 1.921841001 -0.811827655 1.974331355
C -1.901216046 1.828048295 -1.413469526
C 1.252379654 -2.648591870 0.486066412
C 0.002175229 -3.078384064 1.231546630
C -1.291125884 -2.528299748 0.659786413
H -3.411343117 -0.197777722 -1.928677565
H -2.219276294 -1.484788964 -1.857723252
H -3.138891900 0.243964812 0.493539969
H -3.536420371 -1.445391704 0.266473801
H -2.137751569 -3.019798810 1.143135031
H -1.344032016 -2.768007550 -0.400981523
H 0.083971815 -2.883698871 2.297765516
H -0.059621477 -4.164142353 1.145645680
H 2.112953551 -3.200965275 0.869901967
H 1.152671982 -2.897896020 -0.571060654
H 3.358881456 -0.145320690 0.180169639
H 3.359504663 -1.778797214 -0.430804847
H 3.105585094 -0.078580993 -2.274994990
H 1.733494593 -1.176460530 -2.185776555
H 0.434064028 2.128968604 -2.795797026
H 1.291072152 0.835133690 -3.596822660
H -1.262891848 0.604887595 -3.706256801
H -0.458001751 -0.789663268 -2.995987185
H -2.445669910 1.917641041 -0.482677613
H -1.075924962 2.525480001 -1.366853431
H -2.554806799 2.077404746 -2.249620611
H -0.822827122 -0.965469295 2.817804300
H -1.813290957 0.396056279 2.313020178
H -2.567018475 -1.185760016 2.635886807
H 1.114939497 -1.046299440 2.653706862
H 2.814068740 -1.356232073 2.286134674
H 2.099060532 0.256330631 2.021706929
H 2.761377930 2.297912228 -1.690903813
H 1.291133894 2.751965403 -0.791817319
H 2.508517012 1.763624131 -0.013532802
O -0.041321808 1.321537968 0.932563599

^{6}2

Co -0.009146468 0.051659840 -0.025509797
C 2.088027617 2.006809738 -1.112732521
N 1.372272325 0.774807535 -1.548821472
C 0.637788427 1.022513840 -2.827991252
C -0.657426628 0.233760396 -2.847283071
N -1.407597886 0.452988363 -1.579230187
C -2.521107186 -0.523960803 -1.412103180
C -2.821913130 -0.711647929 0.061221463
N -1.591822576 -1.113513157 0.818221491
C -1.787627714 -0.779330289 2.258948625
C 2.297659052 -0.386449782 -1.670041171
C 2.761662834 -0.847219344 -0.302891495
N 1.599644753 -1.169309922 0.596991184
C 1.983063162 -0.877234204 2.010066223
C -1.910417581 1.853004515 -1.468114873
C 1.229848607 -2.615454731 0.423554370
C -0.008202927 -3.085654954 1.176000980
C -1.340992537 -2.579941642 0.640946388
H -3.412081248 -0.179656318 -1.939411276
H -2.219561115 -1.462030112 -1.870674548
H -3.183310838 0.214942054 0.496745402
H -3.604529056 -1.459473061 0.199290389
H -2.155701516 -3.118676907 1.130328374
H -1.403309115 -2.802441005 -0.423082246
H 0.086378367 -2.907485689 2.244266477
H -0.030326558 -4.171712134 1.074156617
H 2.088702140 -3.209741396 0.743643548
H 1.098316456 -2.791496533 -0.644578219
H 3.352533298 -0.076503845 0.179219379
H 3.404231512 -1.722620883 -0.399942623
H 3.160344601 -0.120748041 -2.285702380
H 1.767388690 -1.186505975 -2.184487700
H 0.444314524 2.086764131 -2.906506426
H 1.259236186 0.751981697 -3.682355928
H -1.266826186 0.512536308 -3.709548908
H -0.452543067 -0.833363841 -2.924870011
H -2.479795236 1.964511980 -0.552781875
H -1.081451819 2.548374657 -1.420976358
H -2.543964630 2.088365373 -2.323861367
H -0.917145250 -1.064026989 2.836824057
H -1.929114973 0.291533182 2.360296994
H -2.657927032 -1.307050268 2.651170329
H 1.160360304 -1.092488767 2.679370953
H 2.843090235 -1.483387145 2.297176643
H 2.225365725 0.176132249 2.101377532
H 2.830878192 2.295540630 -1.857645102
H 1.368501808 2.807987479 -0.981759590
H 2.575868573 1.845209106 -0.158720416
O -0.016009882 1.404652831 0.996088574

M06/cc-pVTZ(-f) Cartesian coordinates

^{2}2

Co -0.008733057 -0.118834926 -0.227462607
C 1.968034053 1.979972618 -0.982559667
N 1.317373637 0.736912278 -1.463994732
C 0.630266189 1.005893192 -2.764488487
C -0.684322391 0.274582206 -2.783103162
N -1.383937238 0.499325057 -1.492776978
C -2.536297242 -0.422795163 -1.325320676
C -2.753877402 -0.625737948 0.142481518
N -1.484536242 -1.050937922 0.807104289
C -1.640768403 -0.714796599 2.242942546
C 2.308010467 -0.358315012 -1.587567004
C 2.732662422 -0.775465372 -0.209569461
N 1.531375289 -1.081250187 0.633817759
C 1.858302501 -0.732241230 2.035395942
C -1.826324209 1.905108178 -1.341926113
C 1.239742118 -2.540622767 0.496435540
C 0.002413356 -3.041620102 1.198572940
C -1.297988440 -2.516467022 0.640256801
H -3.426103650 -0.009377546 -1.807361367
H -2.305988490 -1.361015234 -1.832609512
H -3.070259927 0.302877091 0.615875396
H -3.536822970 -1.364495081 0.335578842
H -2.144077083 -3.021266492 1.119052362
H -1.356769137 -2.749390582 -0.429086335
H 0.067735166 -2.890991914 2.276104475
H -0.009778247 -4.126237679 1.072110405
H 2.117862637 -3.081411331 0.865796083
H 1.167889788 -2.761342087 -0.576115517
H 3.294831805 0.018390872 0.276649627
H 3.391483163 -1.646073617 -0.243384375
H 3.166607774 -0.029424582 -2.182174681
H 1.842238994 -1.185688047 -2.132830023
H 0.484130897 2.079587740 -2.860097473
H 1.264725099 0.701498380 -3.599449310
H -1.314097381 0.583691694 -3.623362987
H -0.526618064 -0.805904695 -2.885908249
H -2.348813620 2.027196765 -0.397517006
H -0.976289202 2.579950523 -1.334735043
H -2.493742503 2.163482821 -2.167088685
H -0.832741716 -1.120564258 2.839282483
H -1.655974704 0.364659868 2.357918099
H -2.578938144 -1.143342217 2.605326397
H 1.061521553 -1.021139470 2.710171889
H 2.774022105 -1.247982941 2.335555999
H 2.000079469 0.343833277 2.112643788
H 2.722287181 2.288347172 -1.710669338
H 1.225309432 2.764051046 -0.869557909
H 2.430159830 1.833942564 -0.012649709
O -0.057034488 1.233754899 0.813492262

^{4}2

Co -0.018685786 0.014963688 -0.121729168
C 2.015308885 1.967578443 -0.978202087
N 1.311579746 0.749877791 -1.446719346
C 0.622442463 1.038143693 -2.745950111
C -0.647265539 0.237835626 -2.829244886
N -1.386797887 0.424377162 -1.560659964
C -2.505584664 -0.527924172 -1.386397611
C -2.755158059 -0.661346322 0.086779510
N -1.491130796 -1.032435465 0.799777475
C -1.674749292 -0.693436413 2.230787932
C 2.259239514 -0.381887623 -1.628640676
C 2.726816560 -0.860221221 -0.291616719
N 1.551250669 -1.169674104 0.576978798
C 1.924461319 -0.842432186 1.974926315
C -1.893581211 1.815873157 -1.444649683
C 1.240488796 -2.616963128 0.456004319
C 0.002456655 -3.056223107 1.193981025
C -1.281625457 -2.500782556 0.635831274
H -3.399351214 -0.173658340 -1.907978228
H -2.225754990 -1.479143893 -1.841089163
H -3.101100345 0.280701666 0.509896557
H -3.523585621 -1.408453055 0.303548956
H -2.135204615 -2.994348498 1.112867862
H -1.338574166 -2.731133690 -0.432022753
H 0.087532077 -2.880730552 2.266515016
H -0.059496278 -4.142036153 1.095439082
H 2.108573994 -3.176125108 0.822641324
H 1.137992445 -2.847889819 -0.610209321
H 3.328413007 -0.100262432 0.202693096
H 3.363197699 -1.743098391 -0.389252165
H 3.097194519 -0.058257947 -2.254665226
H 1.737834355 -1.175853664 -2.170636177
H 0.419304513 2.105522042 -2.793463991
H 1.297457411 0.811750242 -3.574633127
H -1.256405708 0.542392736 -3.686397555
H -0.439071251 -0.830719187 -2.939393480
H -2.474227659 1.929812729 -0.534771984
H -1.078770006 2.530133817 -1.395979882

H -2.525462307 2.034946939 -2.309186797
H -0.816183203 -0.997298902 2.819223760
H -1.803681034 0.381074595 2.333197869
H -2.560488573 -1.210279583 2.610183920
H 1.125025861 -1.087421189 2.665008174
H 2.813602627 -1.414232778 2.255154151
H 2.129934340 0.221569401 2.058146933
H 2.746404022 2.273715309 -1.730801879
H 1.292643871 2.764855078 -0.823341701
H 2.518938983 1.785961832 -0.033717961
O -0.050173133 1.321366495 0.939411928

[6]2
Co -0.011461756 0.055491921 -0.011016064
C 2.085649406 1.989500519 -1.133476221
N 1.355485362 0.763916997 -1.530703268
C 0.631548144 0.996088371 -2.806939120
C -0.649607245 0.206019711 -2.817857899
N -1.395521907 0.448881834 -1.563037357
C -2.510882858 -0.513141574 -1.395870539
C -2.803615235 -0.692697242 0.069107049
N -1.577837790 -1.095907678 0.810643583
C -1.791453772 -0.808687337 2.249100464
C 2.283687557 -0.383802250 -1.656058345
C 2.745106416 -0.834859597 -0.297062619
N 1.588159370 -1.152074915 0.591859555
C 1.989506117 -0.898973249 1.997477031
C -1.911537145 1.837349347 -1.505009561
C 1.219836739 -2.587368512 0.400414959
C -0.008672355 -3.059712909 1.142575554
C -1.327122032 -2.550794104 0.608212884
H -3.399062830 -0.159024194 -1.926883767
H -2.222627732 -1.456634445 -1.861051431
H -3.159436343 0.240490349 0.507177172
H -3.590213494 -1.437498502 0.224400101
H -2.154258018 -3.094116606 1.078756001
H -1.380673657 -2.758242335 -0.464165057
H 0.083994858 -2.894546034 2.216516388
H -0.031404794 -4.146440640 1.034999507
H 2.086039675 -3.182561914 0.710344640
H 1.088228465 -2.754089312 -0.674305304
H 3.335711688 -0.058019342 0.184740230
H 3.394037268 -1.710832774 -0.379825601
H 3.143226342 -0.103150935 -2.274690013
H 1.764227409 -1.189800276 -2.181099697
H 0.433371889 2.062274049 -2.899285805
H 1.263868938 0.724869776 -3.656751038
H -1.264781228 0.463292642 -3.686315359
H -0.442205677 -0.867321616 -2.876578672
H -2.505272727 1.975913530 -0.605059769
H -1.096749677 2.555013034 -1.472380338
H -2.534577050 2.032017947 -2.381682499
H -0.922026163 -1.089950953 2.836738943
H -1.968409669 0.255689274 2.385479067
H -2.655094974 -1.371836159 2.612795198
H 1.172496714 -1.108116277 2.681697785
H 2.839886566 -1.534470640 2.258429620
H 2.268099299 0.146612145 2.110505224
H 2.818843209 2.250632532 -1.901323637
H 1.381448952 2.809387288 -1.012384845
H 2.595526890 1.849081575 -0.183728451
O -0.013675824 1.393417062 1.00187440

CASSCF(11,10) Cartesian coordinates

[4]2
x(Å) y(Å) z(Å)
Co -0.088088959 0.032848936 -0.391658630
C -2.695128045 -1.469540695 -1.095293292
N -1.965249252 -0.885358079 0.084060203
C -2.846309305 0.133411320 0.760109329
C -2.017687004 1.262782999 1.375042665
N -1.043987527 1.791353425 0.366905440
C 0.033794214 2.630117405 0.982067013
C 1.283175408 2.574499031 0.105067843
N 1.720338699 1.152072522 -0.148853058
C 2.610250734 1.150055166 -1.363834763
C -1.559569948 -1.964866802 1.046972708
C -0.406116599 -2.785637379 0.478484240
N 0.751279890 -1.903918319 0.077535474
C 1.435414264 -2.528297004 -1.111020662
C -1.757158815 2.575051899 -0.701811293
C 1.718196735 -1.805492061 1.236722933
C 2.878307473 -0.823303737 1.047947054
C 2.496566840 0.659784965 1.055185153
H -0.294340370 3.656471617 1.088446173
H 0.237975108 2.261934955 1.976715809
H 1.091940394 3.038001190 -0.849873222
H 2.089472908 3.127985971 0.570380358
H 3.398691350 1.255808697 1.127935953
H 1.911154175 0.867686682 1.941403997
H 3.471416717 -1.075175119 0.181953382
H 3.538623794 -0.968297984 1.895295595
H 2.121033808 -2.795195755 1.417855295
H 1.152350909 -1.529180724 2.119159680
H -0.730674740 -3.338279981 -0.387068782
H -0.080409058 -3.514825986 1.208625624
H -2.403199173 -2.611215182 1.257573107
H -1.274576771 -1.501173405 1.983178240
H -3.531117553 0.526206374 0.027699112
H -3.446369555 -0.342410172 1.525113466
H -2.674378378 2.049288539 1.727554029
H -1.460479883 0.907640696 2.233232683
H -1.045788507 2.985490816 -1.399514581
H -2.436918038 1.946488414 -1.252240030
H -2.314554504 3.388356765 -0.253986060
H 2.944140008 0.153921357 -1.593991393
H 2.069360890 1.536310897 -2.213067443
H 3.478258198 1.774059598 -1.190588026
H 2.264565115 -1.926938068 -1.438334797
H 1.803932499 -3.514064010 -0.854957042
H 0.736873640 -2.619923115 -1.927965147
H -3.599512048 -1.967345032 -0.768378341
H -2.958832897 -0.681788392 -1.782978662
H -2.076535631 -2.179562144 -1.618180444
O -0.210240281 0.106346858 -2.051301582
